# Diagnostic efficiency of inflammatory signatures to distinguish isolated candidemia from candidemia with bacterial co-infection

**DOI:** 10.3389/fimmu.2025.1692077

**Published:** 2025-10-28

**Authors:** Magdalena Mnichowska-Polanowska, Bartłomiej Grygorcewicz, Barbara Dołęgowska, Agnieszka Boroń, Agata Michnowska, Konrad Jarosz, Magdalena Adamowicz, Bartosz Wojciuk

**Affiliations:** ^1^ Department of Microbiology, Immunology and Laboratory Medicine, Pomeranian Medical University in Szczecin, Szczecin, Poland; ^2^ Department of Genomics and Forensic Genetics, Pomeranian Medical University in Szczecin, Szczecin, Poland; ^3^ Regional Center for Digital Medicine, Pomeranian Medical University in Szczecin, Szczecin, Poland; ^4^ Department of Laboratory Medicine, Pomeranian Medical University in Szczecin, Szczecin, Poland; ^5^ Department of Clinical and Molecular Biochemistry, Pomeranian Medical University in Szczecin, Szczecin, Poland; ^6^ Department of Immunological Diagnostics, Pomeranian Medical University in Szczecin, Szczecin, Poland; ^7^ Clinic of Nephrology, Internal Medicine and Transplantation, Teaching Hospital No 2, Pomeranian Medical University in Szczecin, Szczecin, Poland; ^8^ Clinic of Anesthesiology and Intensive Therapy, Teaching Hospital No 1, Pomeranian Medical University in Szczecin, Szczecin, Poland

**Keywords:** candidemia, critically ill patient, inflammatory response, intensive care, proteomics

## Abstract

**Introduction:**

To identify the differences in inflammatory response between critically ill patients responding to combined bacterial-fungal sepsis and those with isolated fungal sepsis.

**Methods:**

A retrospective case-control study compared ICU patients who were exposed (n=24) and unexposed (n=20) to candidemia. Two exposure modes were analyzed: isolated candidemia (C; n=12) versus candidemia with bacterial co-infection (BC; n=12). Targeted proximity extension assay (PEA) was used to examine differences in serum inflammatory proteome between groups. Differential inflammatory proteins served as input for a logistic regression model to validate their effectiveness in discrimination.

**Results:**

Two major clusters—candidemia cases and controls—were identified based on differential protein expression analysis. In five-fold cross-validation, LAP-TGF beta-1 was identified as the main driver, effectively distinguishing isolated candidemia [AUC 0.95; 95% CI 0.853–1.000]. TRANCE and IL-17C showed potential as a diagnostic signature indicating bacterial co-infection in the context of candidemia.

**Discussion:**

The three-protein logistic regression panel (LAP-TGF beta-1, TRANCE and IL-17C) differentiated cases with isolated candidemia from those with candidemia and bacterial co-infection [AUC 0.82; 95% CI 0.629–0.968]. A three-protein inflammatory signature distinguished isolated fungal sepsis from combined bacterial-fungal sepsis. This study is the first to explore the inflammatory response to differentiate isolated candidemia from candidemia with bacterial co-infection.

## Graphical Abstract

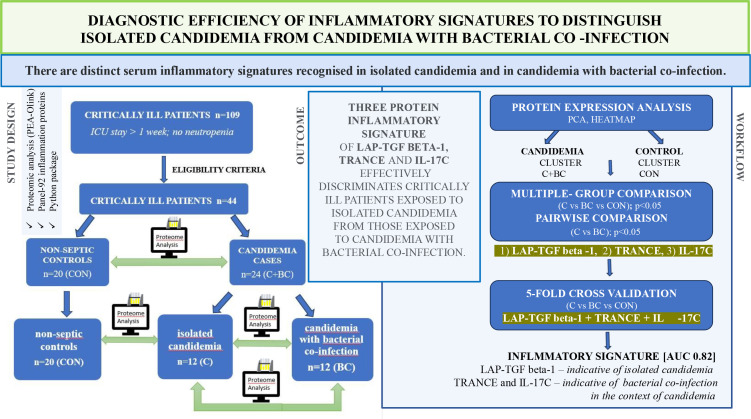

## 1 Introduction

Intensive care unit (ICU) candidemia poses an increasing challenge for modern medicine, as it is a critical and growing condition characterized by high mortality (1–3) and diverse predisposing factors (4, 5). Polymicrobial candidemia, caused by bacterial co-infection (bacteremia), is a predictable risk in ICU patients and often leads to overuse of antifungal prophylaxis. Conversely, isolated candidemia, which typically presents insidiously, results in delayed antifungal treatment. Therefore, there is an urgent need to improve differentiation between isolated candidemia and candidemia with bacterial co-infection. This distinction will help identify patients at higher risk for complications and aid in personalizing therapy. Patients in the ICU with a risk of candidemia must be stratified more precisely before initiating sepsis management, ideally based on specific genomic and proteomic biomarkers involved in antifungal response (6). Currently, identifying patients at high risk for both combined and isolated candidemia mainly relies on clinical risk factors, blood cultures with moderate efficacy, and non-culture assays targeting either non-viable pathogens or pathogen-related biomarkers (7, 8). However, this diagnostic approach appears inadequate, although strict adherence to guidelines may improve patient survival (1, 8, 9). Growing evidence from biomarker-driven strategies and high-throughput ‘omics’ technologies highlights the variety of molecular biosignatures in sepsis (5–7, 10, 11). The imbalance between pathogen clearance and systemic inflammation is a key factor driving septic complications. Therefore, it is critically important to identify clinically relevant biomarkers of immune dysregulation in sepsis (5, 10).

This approach to sepsis, whether of bacterial or fungal origin, viewed as a dysregulated, heterogeneous immune response, underscores pathogen type-dependent changes in the expression of inflammation-related proteins (10, 12). As shown by transcriptome profiling, the cytokine-mediated immune response to candidemia in hospitalized adults differs from the response to bacterial sepsis (13). The emerging stratification of critical patients based on molecular profiling may potentially identify pathogen-specific dysregulation (5, 10, 14).

Accordingly, we employed targeted proteomic profiling in response to candidemia. To date, this targeted proteomic approach to fungal and combined bacterial-fungal sepsis has not been studied in ICU patients. Understanding the inflammation-related proteins expressed during candidemia will help clarify the pathophysiology of this critical condition in the ICU. Additionally, a pathobiology-driven approach offers a starting point to identify protein signatures of distinct host responses to candidemia, whether or not coexisting with bacterial co-infection.

## 2 Material and methods

### 2.1 Study design and patients

**Figure 1:**
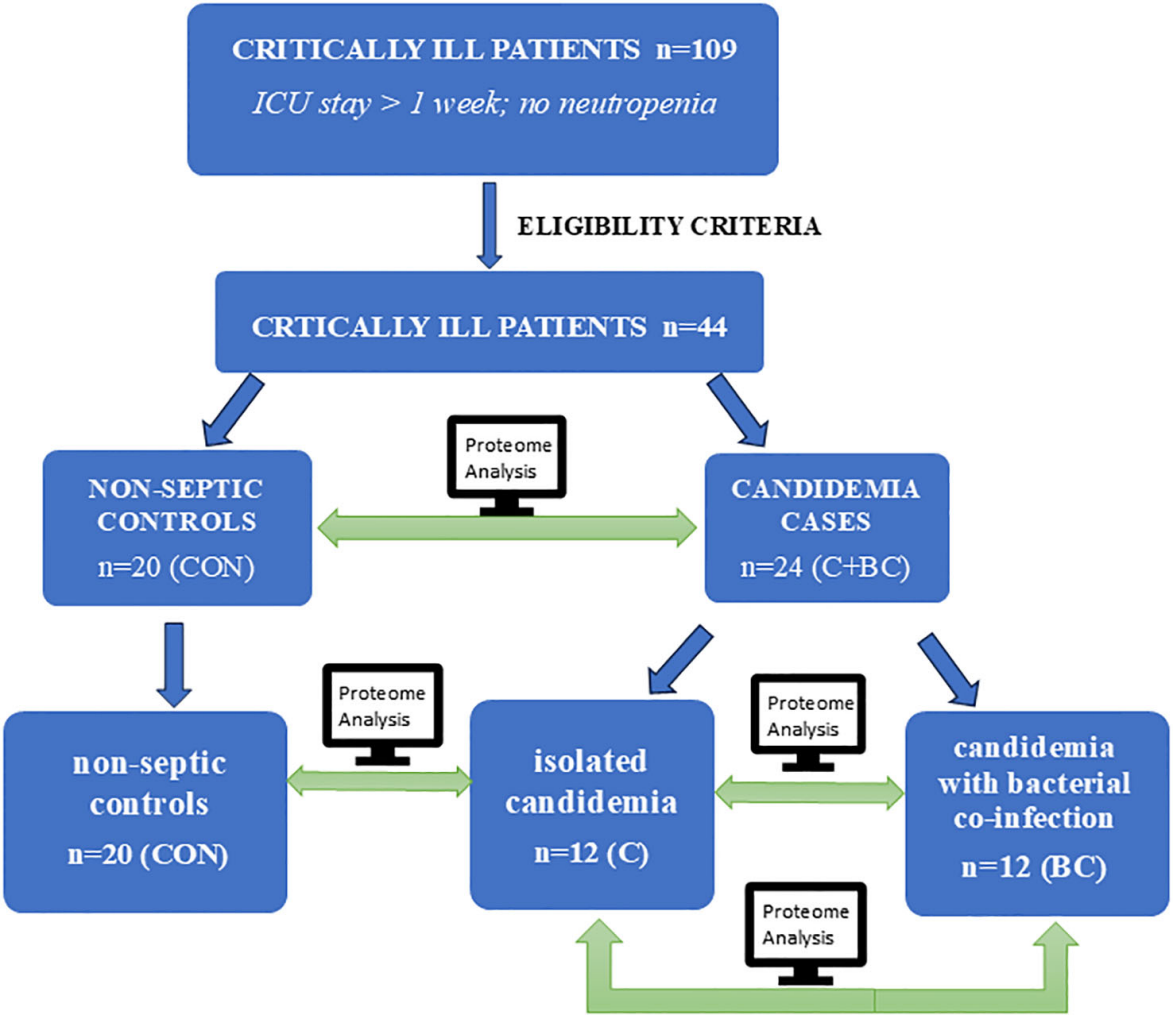
Study design and protein expression analysis workflow across the study groups.

We analyzed the expression profiles of 92 targeted inflammatory mediators in ICU patients with isolated candidemia, candidemia combined with bacterial co-infection, and compared these cases both to each other and to non-septic ICU controls to identify key differences in the serum inflammatory proteome. The study design and the comparative protein expression analysis workflow is illustrated in **Figure 1**. The STROBE (Strengthening the Reporting of Observational Studies in Epidemiology) statement checklist was followed to report this retrospective case-control study. The study protocol adhered to the International Conference on Harmonization–Good Clinical Practice and Declaration of Helsinki guidelines. The study protocol and informed consent form were approved by the Pomeranian Medical University Ethics Committee in Szczecin (approval numbers: KB-0012/279/06/16; KB-0012/150/03/18). Detailed patient medical data were collected from the Electronic Medical Record System in the ICU, anonymized, and are available upon reasonable request and with the hospital’s consent.

#### 2.1.1 Patients and eligibility criteria

The study included 109 critically ill adult participants admitted to the 16-bed ICU of the University Clinical Hospital in Szczecin, Poland, between October 2017 and September 2018. They had ICU stays longer than one week, no neutropenia (<1,5 G/l) throughout their ICU stay, and no history of chronic inflammatory conditions to minimize participant selection bias. All 109 patients were monitored using clinical, laboratory, and microbiological criteria from the day of ICU admission until their death or discharge. Additionally, 44 of these patients met specific exclusion and inclusion criteria (**Supplementary 2.1 Materials and Methods**). They were classified as cases with candidemia variants or controls presenting neither candidemia nor bacteremia. Controls were critically ill patients with high risk of infections, received antibacterial antibiotics in prophylaxis if needed, dependently on the clinical history. The patients’ exposure to candidemia and bacteremia was monitored twice weekly through blood cultures (BACT/ALERT system; bioMérieux) and mannan concentration (PlateliaTM Candida Ag Plus; Bio-Rad) starting at ICU admission. Multifocal Candida colonization, defined as a Candida colonization index of ≥ 0.5, was assessed weekly. Once the bacterial sepsis was confirmed, therapy with broad spectral antibiotics (III generation cephalosporins) was administered and then verified according to antibiogram. When candidemia was confirmed, fluconazole or echinocandins were used due to the patient’s clinical status and antifungal susceptibility testing report. The study tracked all enrolled patients through to the end of their ICU stay. The controls did not develop neither isolated candidemia nor candidemia with bacterial co-infection to the end of their ICU stay.

#### 2.1.2 Defining case and control groups using microbiological data

The participants enrolled as cases represented two modes of exposure to candidemia: isolated candidemia (C; n=12) and candidemia with bacterial co-infection (BC; n=12). This exposure was confirmed through blood culture. Microbiological surveillance revealed *Candida* species growth in one or multiple samples from venipuncture (but not from the catheter). In BC, confirmed bacterial growth preceded a positive *Candida* blood culture. The initial results of mannan were negative, below the threshold for a positive report (<125 pg/ml). A 4–6 fold increase in mannan was observed for both C and BC cases before a positive blood culture. The controls were non-neutropenic, non-septic ICU individuals with neither candidemia nor bacteremia (CON; n=20). Control subjects with negative blood culture outcomes for both *Candida* and bacteria were age-matched to the cases. The negative mannan assessment remained stable throughout the study period in CON.

#### 2.1.3 Definition of case and control groups using clinical data

All candidemia cases were clinically reported as sepsis, diagnosed using Sepsis-3 criteria (12), and met the updated FUNDICU (Invasive Fungal Diseases in Adult Patients in Intensive Care Unit) definition of candidemia (15). No significant differences were observed in sex, age, or the day of candidemia detection between the C and BC subgroups. The organ dysfunction and mortality risks for patients and controls were assessed with SOFA (Sepsis-related Organ Failure Assessment) and APACHE II (Acute Physiology and Chronic Health Evaluation) scales, respectively. The Candida score (CS) was used to evaluate the potential risk of candidemia in individuals meeting the eligibility criteria. CS included clinical sepsis assessment, parenteral nutrition, post-abdominal surgery, and the screening for multifocal Candida colonization (16, 17), with a threshold >3 deemed relevant.

### 2.2 Serum collection for proteome research

Peripheral venous whole blood (7.5 ml) was collected into CATs (Clot Activator Tubes) from each candidemia case and non-septic control to obtain serum samples for targeted proteomic profiling of the inflammatory response to candidemia. Serum samples from candidemia cases were received within one hour of the first positive blood culture report for *Candida* and before the administration of echinocandins. No neutropenia (<1,5 G/l) was observed in patients at the time of blood sampling for proteomics. Serum samples from non-septic controls enrolled in the study were matched with cases based on the sampling time point. Both case and control whole blood and serum samples were processed according to the same protocol. After blood clotting within 30 minutes at room temperature, serum samples were separated by refrigerated centrifugation at 2000 x g for 10 minutes, immediately aliquoted into clean, secure lock tubes (Eppendorf, Poland), and stored at -80 °C until proteomic analysis.

### 2.3 Inflammatory protein profiling

Case and control serum samples were conditioned with one freeze-thaw cycle to eliminate sample handling bias. Inflammatory protein levels were quantified using a multiplex proximity extension assay (PEA) with the Olink^®^ Target 96 Inflammation Panel, with all serum samples processed in a single batch to minimize assay variability (18). The following panel data—protein name, assay abbreviation, Olink ID, and UniProt number—are available in **Supplementary Table 1**. The 96-well PCR (polymerase chain reaction) plate was loaded with 1µl of serum from each patient in duplicate and tested using the standard protocol provided by the Olink supplier (ALAB Laboratories, Warsaw, Poland)—testing procedure rules are described in **Supplementary 2.3 Materials and Methods**.

### 2.4 Differential protein expression analysis

The protein expression data were first normalized to account for variability in measurement scales. The dataset included Normalized Protein eXpression (NPX) values for 92 cytokines, presented on a log 2 scale, for different patient groups. To reduce dimensionality and visualize data variability between groups (candidemia vs. control), we performed principal component analysis (PCA) based on NPX data and visualized the results. Then, hierarchical clustering was performed to identify patterns and clusters within protein expression data, which were visualized in a heatmap.

Differential expression analysis of inflammatory proteins between groups (C, BC, and CON) was conducted with multiple comparison correction using the Benjamini- Hochberg false discovery rate (FDR). Pairwise comparisons among groups (CON, BC, and C) were assessed using Welch’ s t- test, suitable for unequal variances, to identify differentially expressed proteins (DEPs). Volcano plots were generated to visualize DEPs. Serum NPX concentrations of LAP- TGF beta- 1, TRANCE, and IL- 17 C were quantified in three predefined clinical groups—BC, C, and CON—and all subsequent analyses were performed in Python (v 3. 11) with scikit- learn (v 1. 4). For each of the three binary contrasts of interest (BC vs. CON, C vs. BC, C vs. CON), the dataset was restricted to the two relevant groups, after which missing marker values were replaced by the column median, and each variable was z- standardized; both steps were implemented within a scikit- learn Pipeline to ensure parameters were estimated solely from the training folds. Discrimination based on a single marker was modeled with logistic regression (solver = “liblinear”, max _ iter = 1000), while the three- protein panel was fitted with LogisticRegressionCV using an elastic- net penalty (l 1 _ ratio = 0.5. 5) and ten candidate values of the regularization parameter C; tuning was nested within the outer cross- validation scheme. Predictive performance was evaluated with stratified five- fold cross- validation that preserved class balance and used a fixed random seed for reproducibility. For each fold, out- of- fold class probabilities were collected with cross _ val _ predict, aggregated across folds, and analyzed via ROC analysis (roc _ curve and auc). Confidence limits were obtained through non- parametric bootstrap resampling of the out- of- fold prediction matrix (1, 000 replicates, resampling observations with replacement while ensuring the presence of both classes), from which 95% percentile intervals were derived for both the global AUC and the entire ROC curve. All reported AUCs thus provide unbiased estimates of generalizable diagnostic accuracy, and all intervals account for both sampling variability and model- selection uncertainty.

### 2.5 Statistics

All statistical analyzes and dataset visualizations were performed using Python, with packages such as Pandas, NumPy, SciPy, scikit-learn, statsmodels, matplotlib, and seaborn. A p-value of less than 0.05 was considered statistically significant for all tests. Continuous variables were reported as means or medians, depending on their distribution. Categorical variables were presented as counts and percentages and compared using the Chi-squared test. Non-normally distributed continuous variables were compared using the Mann-Whitney U test. A more detailed description of the statistical approach is attached (**2.4 Supplementary Materials and Methods**).

## 3 Results

### 3.1 Clinical and laboratory variability among patients with candidemia in the ICU

SOFA and APACHE II scoring systems could not distinguish subgroups of candidemia cases. A Candida score >3, PCT >2 ng/ml, and ICU mortality were significantly more common in the BC subgroup than in the C subgroup. *Candida* (C.) non-*albicans*-derived candidemia caused by azole-resistant species (mostly *C. glabrata*) was more frequently identified in the BC group. Baseline characteristics between candidemia subgroups are summarized in **Table 1**.

**Table 1:** Baseline characteristics of patients with isolated candidemia (C), candidemia with bacterial co-infection (BC), and non-septic controls with neither candidemia nor bacteremia (CON), hospitalized in the intensive care unit.

Variables	ICU controls	ICU cases		p-value
	Non-sepsis (CON)	Isolated Candidemia (C)	Candidemia with bacterial co-infection (BC)	
Patient’s data				
Number of participants (*n)*	20	12	12	NA
Sex, male/female (*n)*	11/9	4/8	8/4	0.202
Age (*years, median*, *(IQR))*	67.5 (57-75.2)	66.0 (58.2 -73.0)	67.0 (63.0-69.0)	0.863
DCD -day of candidemia detection/(sampling day for proteomics) *(DCD median, (IQR))*	NA (14)	14/(14) (10.8-16.5)	16/(16) (11.8-19.5)	0.301
Clinical data				
SOFA score (*points, median (IQR))*	9 (7-10.2)	7 (6-8.2)	9 (8-12)	0.306
APACHE II (*points, median, (IQR))*	20.0 (15-23)	20.5 (19.2-23.2)	23.5 (18.7-29.7)	0.158
ICU mortality n (%)	1 (5.0)	3 (25.0)	8 (66.6)	**0.015**
Sepsis developed in ICU - positive blood culture *n (%)*	0 (0.0)	12 (100%)	12 (100%)	NA
Abdominal surgery in ICU *n (%)*	2 (10.0)	2 (16.7)	6 (50.0)	**0.032**
Total parenteral nutrition *n(%)*	4 (20.0)	3 (25.0)	4 (33.3)	0.581
Candida score >3; *n (%)*	0 (0.0)	6 (50.0)	11 (91.7)	**0.001**
Laboratory – biochemical data				
CRP >100 mg/dl *n (%)*	1 (5.0)	7 (58.3)	8 (66.7)	0.281
CRP (*mg/dl, median (IQR))*	NA	159.5 (71.7-175.5)	210.8 (94.0-281.5)	0.281
PCT>2ng/ml				
at ICU admission; *n (%)*	0 (0.0)	1 (8.3)	5 (41.7)	**0.025**
2^nd^ week of ICU stay; *n (%)*	0 (0.0)	2 (16.7)	5 (41.7)
PCT *(ng/ml) (median (IQR))*	0.68 (0-8)	0.99 (0.1-5.2)	7.47 (0.1-51)	**0.025**
Laboratory-microbiological data				
Multifocal Candida colonization (colonization index ≥ 0.5) at ICU admission; *n (%)*	0 (0.0)	1 (8.3)	1(8.3)	0.561
2^nd^ week of ICU stay; *n (%)*	7 (35.0)	6 (50.0)	7 (58.3)	0.612
Candidemia etiology				
NAC; *n (%)*	–	6 (50.0)	10 (83.3)	**0.041**
CA; *n (%)*	–	6 (50.0)	2 (16.7)
Azole-resistant candidemia; *n (%)*	–	2 (16.7)	9 (75.0)	**0.002**

NA,not applicable; p-values highlighted in bold indicate statistically significant differences (p-values <0.05) between the C and BC groups; SOFA, Sequential Organ Failure Assessment; APACHE, Acute Physiology and Chronic Health Evaluation; CRP, C reactive protein; PCT, procalcitonin; NAC; non-albicans Candida; CA; C. albicans; For the comparison between groups: 1) Chi-squared test was used for categorical variables (sex, ICU mortality, DCD, sepsis in ICU, abdominal surgery, total parenteral nutrition, multifocal Candida colonization, candidemia etiology, azole-resistant mediated candidemia), whereas 2) Mann-Whitney U test was provided for continuous non-parametrical variables (age, SOFA, APACHE II, Candida score, CRP, PCT).

### 3.2 Distinction between candidemia cases and non-septic controls in ICU

**Figure 2:**
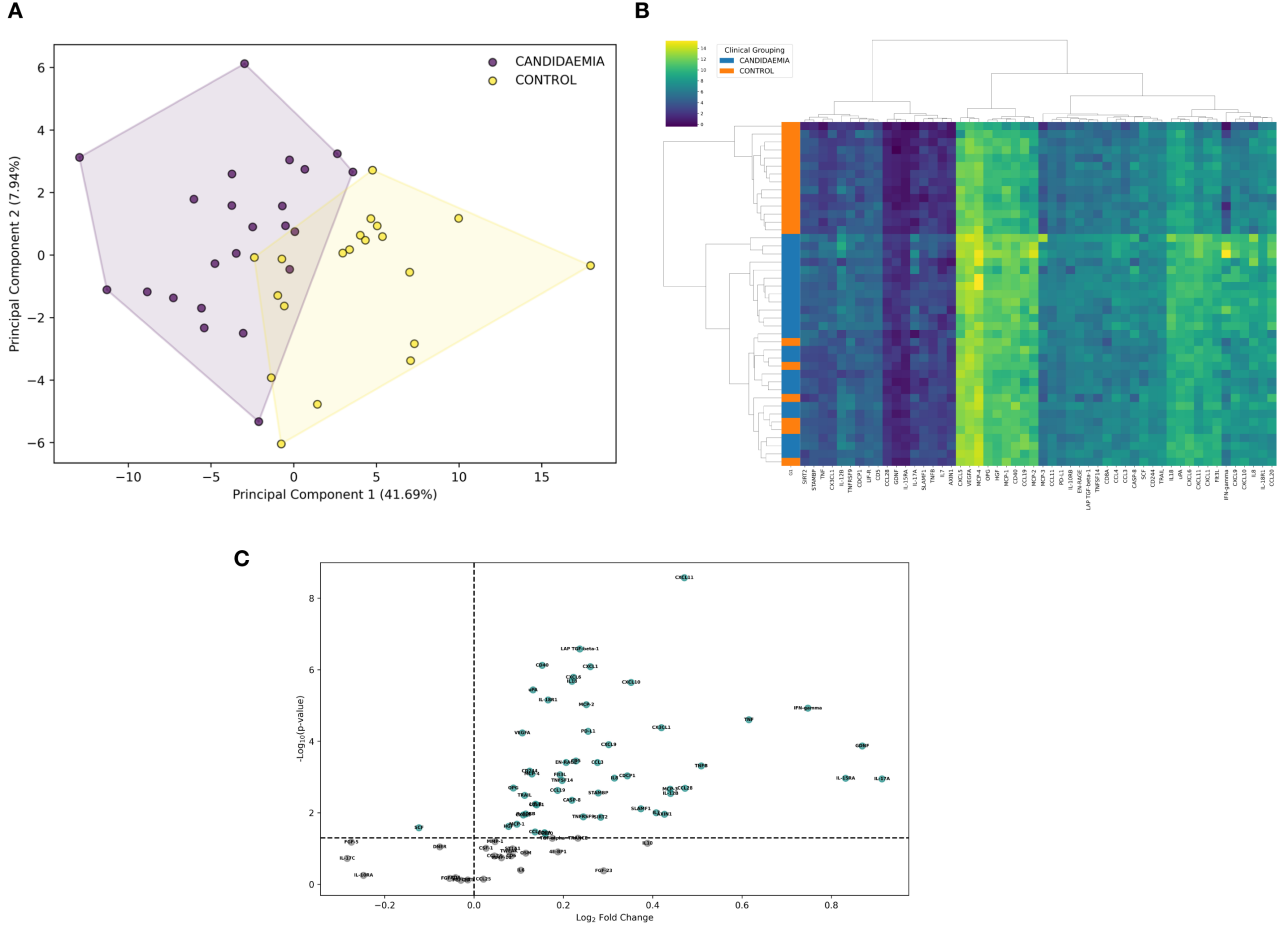
Comparison of inflammation-related proteins in ICU patients exposed to candidemia (cases) and unexposed to candidemia (controls). Differential analysis was based on NPX (Normalized Protein eXpression) to differentiate between study groups. **(A)** Principal component analysis (PCA), visualized with convex hulls, represents the variation of inflammatory proteins among study groups. The x-axis shows the first principal component (PC1), which explains 41.69% of the variance, while the y-axis shows the second principal component (PC2), explaining 7.94% of the variance. The legend indicates the candidemia and control cases, differentiated by color and based on NPX variation. Each dot represents a single ICU patient, positioned in the PCA plot according to the NPX profile. **(B)** Heatmap highlights differences in differentially expressed proteins (DEPs) between candidemia cases and controls. Hierarchical clustering was performed using Ward’s method with Euclidean distance; adjusted p-value <0.05. Clusters are labelled as 1 (control cluster), 2a (homogeneous candidemia cluster), and 2b (heterogeneous candidemia cluster). Each row represents a patient, and each column an individual protein. The color scale from dark blue to yellow indicates low to high protein expression levels. ICU patients are grouped by diagnosis (candidemia vs. control), as shown by the color bar on the left. **(C)** Volcano plot displays the log2 fold change plotted against –log10 p-value for proteins detected by the OLINK Inflammatory Panel between candidemia cases and controls unexposed to candidemia; 52 significantly dysregulated (up- and downregulated) proteins are highlighted in green, indicating differences in protein abundance between groups; adjusted p-value <0.05. scheme: particular figures for this panel attached as separate files.

Out of the 92 inflammation-related proteins analyzed by Olink’s PEA, 75 (82%) were detected above the low limit of detection (LLOD) in more than 75% of the samples and were used as variables in PCA (**Supplementary Table S2**). NPX profile-based PCA revealed two clusters, clearly distinguishing candidemia cases (C+BC subgroups) from non-septic controls (**Figure 2A**). Although there was minimal overlap between the clusters, proteomic profiling-based PCA successfully separated the candidemia cases from non-septic controls, accounting for nearly 50% of the variance.

Although the NPX-profile-based PCA separated candidemia cases from non-septic controls, differential expression analysis identified 52 DEPs in candidemia cases (C+BC subgroups) out of 75 detected proteins (p < 0.05), compared to controls (**Supplementary Table 2**). Incorporating these 52 high-quality DEPs into hierarchical clustering, we divided the patients into two major clusters (n=13 and n=31) – cluster 1, consisting exclusively of non-septic controls, and cluster 2, mainly composed of candidemia cases. Within cluster 2, a homogeneous candidemia subcluster 2a (cases) and a heterogeneous candidemia subcluster 2b (cases and 7 controls) were identified. Cluster 1 showed lower relative protein abundance than the entire cluster 2, while subcluster 2a exhibited the highest protein expression among all groups. The representative protein expression heatmap is shown in **Figure 2B**.

### 3.3 Proteome profiling between candidemia cases and non-septic controls (C+BC vs controls)

Among the 52 DEPs, 51 were found upregulated, and 1 protein was downregulated in candidemia cases compared to controls (**Supplementary Table 3**). The proteomic profile in candidemia (**Figure 2C**) included eight of the most significantly upregulated DEPs: CXCL11, LAP-TGF beta-1, CD40, CXCL1, CXCL6, IL18, CXCL10, and uPA. IL-17A was inversely regulated compared to IL-17C.

### 3.4 Proteomic profiling in particular subgroups of candidemia cases versus non-septic controls (BC vs CON and C vs CON)

High numbers of proteins were upregulated in the C and BC subgroups compared to controls: 46 and 44, respectively (**Supplementary Table 4** and **Supplementary Table 5**). Among these, 35 were overlapped. The highest upregulated expressions in isolated candidemia (**Supplementary Figure 1A**) included CXCL6, LAP-TGF beta-1, CXCL1, CXCL11, and CD5, while in candidemia with bacterial co-infection, CXCL11, CD40, uPA, CXCL1, CXCL10, and IL-18 were the most abundantly upregulated (**Supplementary Figure 1B**). Proteins with uniquely altered expression in each subset are presented in **Table 2**.

**Table 2:** Qualitative differences in DEPs within subgroups of candidaemia cases when compared to non-septic ICU controls (CON); DEP (differentially expressed proteins by OLINK Inflammatory Panel); BC subgroup (candidaemia with bacterial co-infection); C subgroup (isolated candidaemia)—assay abbreviations of proteins available in **Supplementary Table 1**.

C subgroup vs CON	BC subgroup vs CON
Unique up- regulated DEPs	
TRANCE	CCL4
TWEAK	IL-10
SIRT2	CCL19
AXIN1	IL-10RB
LIF-R	MCP-1
CD244	HGF
MMP1	TNFRSF9
CASP-8	MCP-3
CXCL5	IL-7
CD8A	
CD6
Unique down- regulated DEPs	
IL-17C	-
SCF	-

**Figure 3:**
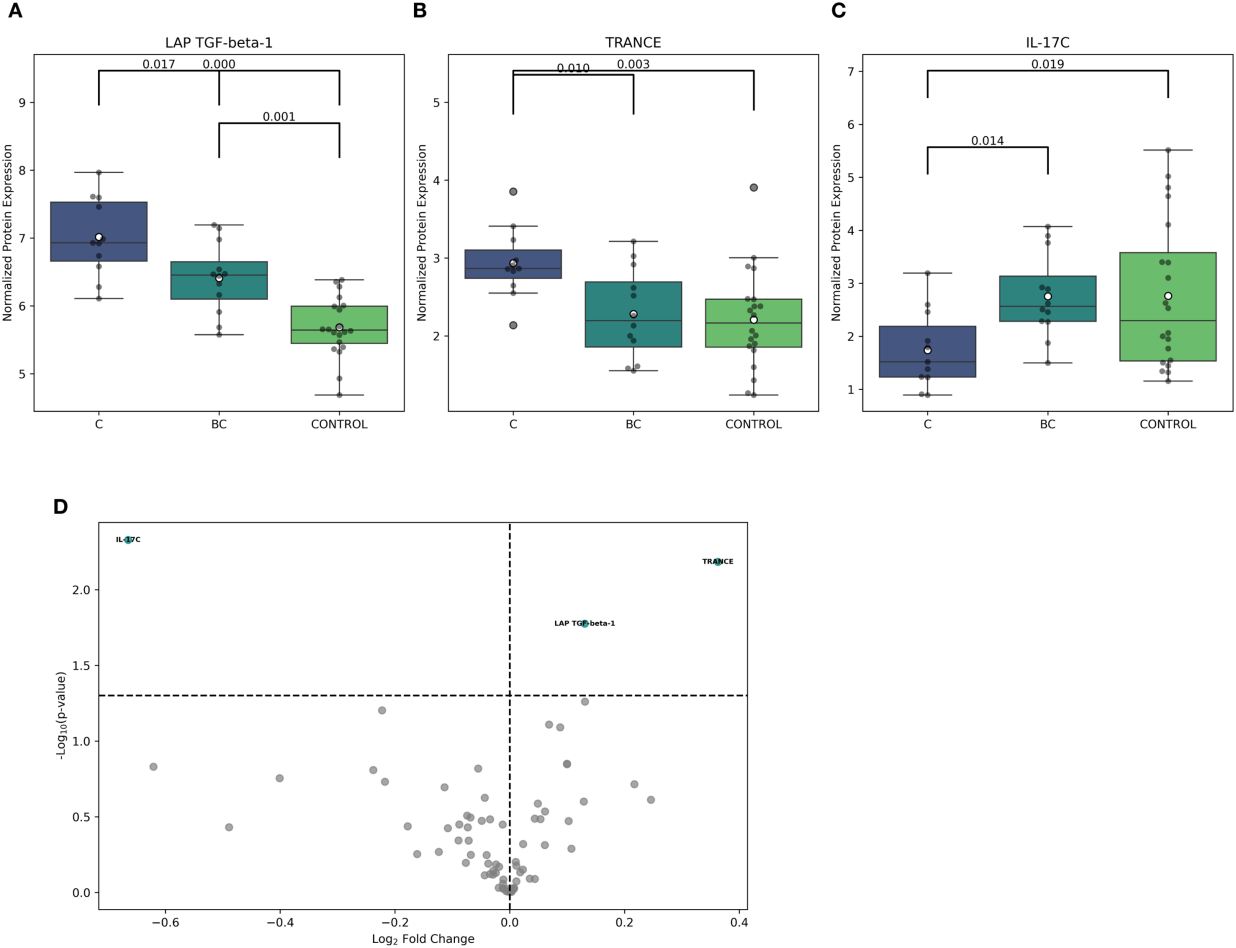
Comparison of inflammation-related proteins in ICU patients exposed to isolated candidemia and candidemia with bacterial co-infection. Differential analysis was based on NPX (Normalized Protein eXpression) to distinguish between study groups. **(A–C)** The following boxplots show significant differences in LAP-TGF beta -1, TRANCE, and IL-17C protein expression between isolated candidemia **(C)**, candidemia with bacterial co-infection (BC), and non-septic ICU controls, who were exposed to neither candidemia nor bacteremia (CON), using multiple testing with ANOVA (Analysis of Variance), corrected by Benjamini-Hochberg to control the false discovery rate (risk of false positive results). **(D)** Volcano plot displays the log2 fold change against –log10 statistical p-value for proteins detected by the OLINK Inflammatory Panel after exposure to isolated candidemia and candidemia with bacterial co-infection. The differentially expressed proteins, up- and downregulated, are highlighted in green, showing protein abundance between groups (adjusted p-value <0.05). scheme: particular figures for this panel attached as separate files.

Furthermore, the multiple-group comparison showed significant differences in LAP-TGF beta-1, TRANCE, and IL-17C expression among C, BC candidemia subgroups, and controls. LAP-TGF beta-1 expression was notably different across all groups, with the highest levels in group C (**Figure 3A**). Similarly, TRANCE (now updated to TNFSF11) was significantly upregulated in group C compared to the other two groups (BC and CON) (**Figure 3B**). Conversely, IL-17C expression was significantly lower in group C and set this group apart from BC and CON (**Figure 3C**). However, the range of IL-17C expression measured by NPX values was highly variable in the controls.

Using pairwise comparison, three differentially regulated inflammatory proteins were identified in C and BC candidemia subgroups. Cases with isolated candidemia showed a significant upregulation of TRANCE (p=0.006) and LAP-TGF beta-1 (p=0.01), along with a notable downregulation of IL-17C (p=0.004) (**Figure 3D**).

### 3.5 Five-fold cross-validation of proteomic profiling data in distinguishing between the study groups

**Figure 4:**
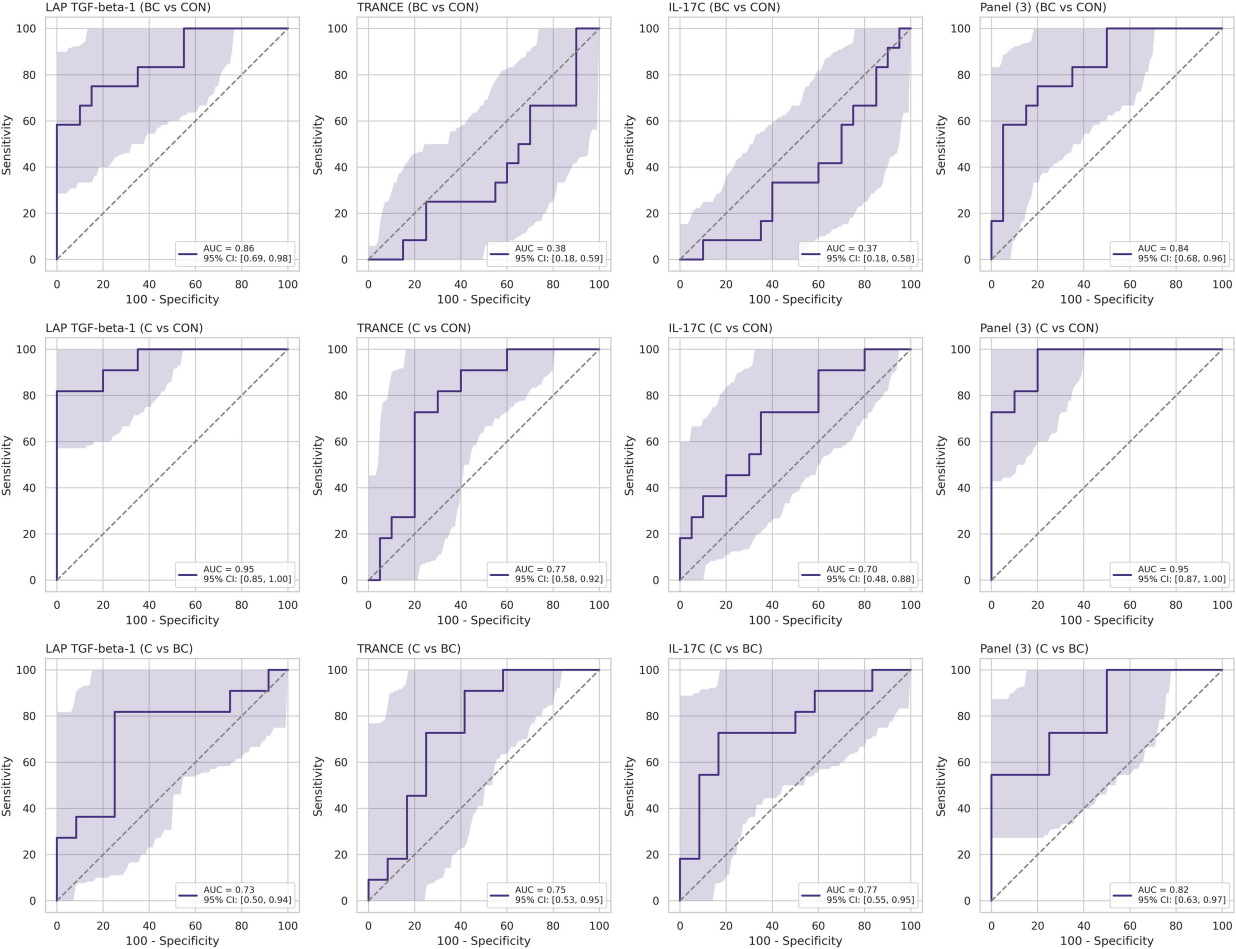
Cross-validated receiver-operating-characteristic (ROC) curves for individual serum proteins and the three-analyte panel. Each subplot displays the discriminatory performance of a single protein—LAP-TGF beta-1, TRANCE, or IL-17C—or the three-protein logistic regression panel [Panel (3)] for a specific clinical contrast: BC vs CON (top row), C vs CON (middle row), and C vs BC (bottom row).

Using stratified five-fold cross-validated logistic regression, we assessed the diagnostic effectiveness of three serum proteins—LAP-TGF beta-1, TRANCE, and IL-17C—across three clinical groups (BC, C, CON). In the BC versus CON classification, LAP-TGF beta-1 emerged as the primary discriminator, achieving an AUC of 0.86 with a 95% bootstrap confidence interval (CI) of 0.69–0.98; the three-protein panel did not significantly improve performance (AUC 0.84, CI 0.68–0.96), with TRANCE and IL-17C performing at chance level (**Figure 4**, top row). In distinguishing between C and CON, LAP-TGF beta-1 alone achieved a high AUC of 0.950 (CI 0.85-1.00); the combined three-protein panel produced a comparable AUC of 0.955 (CI 0.87-1.00), while TRANCE and IL-17C minimally contributed to the diagnostic accuracy (**Figure 4**, middle row). In the C versus BC comparison, individual proteins showed only moderate classification ability (IL-17C – AUC 0.77; TRANCE – AUC 0.75; LAP-TGF beta-1 – AUC 0.73, all with broad and overlapping CIs). Multivariate modeling was necessary to effectively distinguish between C and BC, with the combined three-protein panel reaching an AUC of 0.82 (CI 0.63-0.97) (**Figure 4**, bottom row). TRANCE and IL-17C unexpectedly contributed to the model’s performance by indicating bacterial co-infection in the context of candidemia.

## 4 Discussion

Indeed, there is a growing challenge in distinguishing between combined bacterial-fungal sepsis and isolated fungal sepsis. In this study, targeted proteome profiling explored a diverse host response in cases of culture-proven candidemia in the ICU. To date, proteome profiling in *Candida*-stimulated PBMCs from healthy volunteers has been reported (19). This case-control study showed that unique inflammatory serum proteomes are identified in both isolated candidemia and candidemia with bacterial co-infection. A protein expression signature that is exclusively characteristic of isolated candidemia holds significant promise for enhancing clinical decision-making.

Unique serum protein profiles were identified in isolated candidemia and in candidemia combined with bacterial sepsis. Proinflammatory chemokines, INF-γ and IL-17A, were found to be significantly upregulated in both groups with candidemia compared to controls. Our findings establish LAP-TGF beta-1 as the main marker indicating isolated candidemia in ICU patients. However, this panel of three proteins shows potential for distinguishing bacterial co-infection in patients with candidemia. To the best of the authors’ knowledge, this is the first study using targeted proteomics to compare inflammatory protein profiles in ICU patients with isolated candidemia versus those with candidemia and bacterial co-infection. Therefore, comparing these results with other similar studies is challenging. Chemokines and IL-17A upregulation in all candidemia cases align with current understanding of immunity against *Candida* (20). CXCL11 and CXCL10, which are highly upregulated, are IFN-γ-induced chemokines involved in antifungal Th1-mediated immunity. CXCL1 and CXCL6 promote phagocytosis by attracting neutrophils, which are essential defense against *Candida* blastospores. Transcriptome studies (13) highlight the importance of neutrophil activation in clearing *Candida* spp. and reveal a distinct gene expression response in candidemia compared to acute bacterial infections. Nonetheless, neither chemokines nor IL-17A appeared to differentiate between bacterial/fungal co-infection and isolated candidemia.

Nevertheless, the importance of IL-17 is especially notable. The interaction between IL-17A and IL-17A receptor (R) signaling in both hematopoietic and nonhematopoietic cells is protective against different stages of disseminated candidiasis (21, 22). *In vivo* expression of IL-17A protected normal mice from the lethal dose of *C. albicans*, whereas IL-17AR knockout mice had significantly reduced survival during systemic fungal infection (22). Mice-deficient in IL-17AR signaling in nonhematopoietic cells had increased susceptibility to systemic candidiasis (21). The competence of IL-17 signaling is emphasized in the functional competence of NK cells in fungal infection and, consequently, in protection against disseminated candidiasis (23).

In our study, IL-17A was upregulated in candidemia, but no significant differences in IL-17A levels were observed between candidemia subgroups concerning bacterial co-infection. The IL-17 family consists of IL-17A to IL-17F; the roles of IL-17A and IL-17C cytokines have been of great interest in the context of candidemia. Interestingly, IL-17A expression was notably increased, while IL-17C was significantly decreased in candidemia, indicating distinct functions for different IL-17 cytokine family members in this condition. A similar observation was made by Huang et al. (24), who also proposed different cellular sources for IL-17A and IL-17C. While IL-17A was mainly produced by innate immune cells such as innate lymphoid cells (ILCs), γδ T cells, and NKT cells, IL-17C was expressed in kidney-resident epithelial cells (24). Since 2016, due to its cooperation with other inflammatory cytokines, IL-17C has been regarded as a critical mediator and harmful amplifier of hyperinflammation, contributing to worse outcomes in systemic fungal infections (24).

In our study, the expression of the IL-17C protein varied between the two subgroups of candidemia, with a significant reduction in isolated candidemia. Simultaneously, there was no difference between controls and patients with combined bacterial/fungal sepsis. The animal model indicated that IL-17C-deficient mice infected with *C. albicans* had increased survival, which was linked to decreased production of other proinflammatory cytokines compared with wild-type control mice (24).

Our findings indicate that candidemia affects the IL-17 cytokine family differently than bacterial sepsis. Bacterial infection triggers both IL-17A and IL-17C, whereas candidemia exhibits a contradictory pattern, upregulating IL-17A and downregulating IL-17C. This likely explains the poor performance of IL-17C in the logistic regression model BC vs CON. Conversely, IL-17C alone (being downregulated in isolated candidemia) performed only moderately as a negative discriminator between C and BC. Paradoxically, these two analyses suggest that IL-17C may be an indicator of bacterial and fungal co-infection in the context of candidemia.

In other studies (10, 11), using host-based targeted biomarkers derived from different proteomic profiles, severely ill patients were classified into (endo)types of immune dysregulation, regardless of the etiologic agent. Based on the above criteria (10), biomarkers of sepsis dysregulation that support pathogen clearance (such as IFN-γ and CXCL 10-11) were suppressed, while those promoting systemic inflammation (IL-6 and IL-8) were elevated. In this model, the simultaneous upregulation of CXCL 10–11 and downregulation of IL-17C in isolated candidemia, suggests a more effective clearance and a less dysregulated immune response compared to bacterial sepsis. Similarly it was observed in our study, however the possible impact of immune regulation on the clinical outcomes in candidemia remains hypothetical.

LAP-TGF beta-1 expression varied significantly across all targeted groups; it was considerably higher in both septic cases than in non-septic controls, with the highest levels observed in isolated candidemia. TGF beta-1 is a pleiotropic cytokine that acts in a context-dependent manner (25). It mediates its powerful regulatory effects during inflammatory conditions—either as the beta1 isoform or as a soluble LAP-TGF beta-1. Currently, it remains unclear whether the massive release of TGF beta-1 is triggered by DAMPs released during sepsis or by fungal PAMPs. TGF beta-1 inhibits the development of Th1 cells and regulates adaptive immunity (26). Additionally, TGF beta controls Th17 activity (26). Dectin-1-activated fungal beta-glucan induces TGF secretion and subsequent activation of Th17 lymphocytes (27). This process promotes local mucosal immunity by stimulating the release of proinflammatory IL-17 (27). Serum levels of TGF-beta and IL-17 were significantly higher in the candidemia group compared to bacteremia (28).

The pleiotropy of LAP-TGF beta-1 potentially complicates understanding its role in candidemia. However, as reported in the logistic regression model, aside from the upregulation in both targeted groups, it was also identified as the major discriminative factor between isolated candidemia, candidemia with bacterial co-infection, and non-septic controls. Therefore, we hypothesize that LAP-TGF beta-1 may contribute to immune regulation triggered during isolated candidemia. However, it remains unclear whether these are directly fungal PAMPs as the major drivers of LAP-TGF beta-1 activity or a consecutive activation of different cells, cytokines and receptors. TRANCE is a member of the TNF ligand superfamily, also known as TNFSF11 (18) or RANKL (receptor activator nuclear factor κB ligand) (29–31). Other proinflammatory cytokines can regulate its expression. Activated T lymphocytes produce TRANCE and facilitate signal transduction between antigen-presenting cells and activated T lymphocytes (29). TRANCE (RANKL) is involved in RANK/RANKL signaling pathways responsible for regulating dendritic cell survival, maintaining lymphopoiesis, preventing dysregulated inflammatory responses, and tissue dysfunction (29–31). RANK, the signaling receptor for RANKL (TRANCE), is expressed in antigen-presenting cells such as macrophages and dendritic cells (29). The receptor RANK, in conjunction with ligand RANKL (TRANCE), suppresses the activation of the TLR4 signaling pathway, resulting in a more effective and antigen-specific immune response (30). Inhibition of pathogen-dependent TLR4 signaling via RANK-RANKL interactions may lead to reduced cytokine release and inflammation, thereby preventing organ dysfunction in sepsis (30). Also, recent studies (31) have indicated that RANK-RANKL interactions contribute to the pathogenesis of candidiasis. Our analysis showed that TRANCE expression varied - it reached the highest levels in isolated candidemia cases but did not significantly differ between controls and bacterial co-infections. This was reflected in the logistic regression model, where TRANCE performed less effectively than LAP-TGF beta-1 and was essentially at chance level in distinguishing between bacterial co-infection and controls. This suggests that TRANCE could serve as a negative indicator for bacterial co-infection. We hypothesize that the upregulated expression of TRANCE may possibly promote an effective and less dysregulated immune response in isolated candidemia compared to candidemia with bacterial co-infection.

The current diagnostic protocol to identify patient with candidemia incorporates risk factors, that are present in most patients admitted to the ICU. As a result, virtually, every patient hospitalized for more than one week is considered at risk for developing candidemia. Two main approaches are currently applied: overuse of prophylaxis with antifungals or a ‘watch and wait’ strategy relaying on delayed parameters such as blood culture or serological markers. In clinical trials, cytokines (INF-gamma or GM-CSF or IL7) are being tested as immune modulating therapeutics in sepsis to improve patient’s immune function (26). An approach addressing the combination of the three-proteins: LAP-TGF beta-1, TRANCE and IL-17C represents the potential for diagnostic stratification of septic patients, thus boosts the efficiency of personalized therapeutic strategy. This integrative approach towards the panel of proteins enables a clear identification of specific immune (endo)types occurring in the course of candidemia and can be possibly involved in monitoring protocol in ICU. Such a protocol would take into account not only the pathogen itself, but also the host response, which may vary individually.

It should be noted that our study has several limitations. It reflects a single-center experience. Second, the relatively small number of patients met the inclusion criteria, although this is a well-recognized issue in studies focusing on systemic fungal infections. The significance of the three-protein inflammatory signature (LAP-TGF beta-1, TRANCE, IL-17C) in distinguishing between isolated and combined candidemia needs to be validated in large-sample studies, on another independent cohort to demonstrate whether the identified three-protein inflammatory panel can be commonly applied in clinics. While our findings appear relevant for the both *C. albicans* and the most prevalent non-*albicans* species included in this study, further research is required to establish whether they have broader pan-fungal implications. Despite the high detectability of the targeted proteins and the large number of analytes included in the panel, this analysis remains targeted. Although more challenging, other, possibly untargeted proteomic assays should also be considered reliable for biomarker tracking.

In summary, serum protein profiles differ between patients exposed to candidemia and those unexposed, all managed in the ICU. This difference is independent of bacterial co-infection. Chemokines and IL-17A are significantly upregulated during isolated candidemia compared to controls but do not distinguish it from bacterial co-infection. In contrast, IL-17C is markedly downregulated in isolated candidemia, potentially affecting clinical outcomes. TRANCE and LAP-TGF beta-1 levels are significantly higher in isolated candidemia than in cases of combined sepsis. Although LAP-TGF beta-1 appears to be the primary driver in recognizing isolated candidemia, TRANCE negatively indicates bacterial co-infection. Nonetheless, this three-protein inflammatory signature—LAP-TGF beta-1, TRANCE, and IL-17C—effectively differentiates critically ill patients with bacterial co-infection within the context of candidemia. The cross-validated, bootstrap-based workflow offers an unbiased and reproducible discriminatory efficiency. Therefore, the three-protein inflammatory signature identified by proteomics stratifies critically ill patients with isolated candidemia versus those with candidemia and bacterial co-infection; further study and validation are required for optimal candidemia management.

## Data Availability

The datasets presented in the study can be found in online repository. The name of the repository is: “Targeted proteomics in cadidaemia” and accession number can be found below: https://repod.icm.edu.pl/dataset.xhtml?persistentId=doi:10.18150/SDR3NQ.
