# Supplementary material for: Diagnostic efficiency of inflammatory signatures to distinguish isolated candidemia from candidemia with bacterial co-infection
Source: Front Immunol. 2025 Oct 28;16:1692077. doi: 10.3389/fimmu.2025.1692077 (PMC12602200; doi:10.3389/fimmu.2025.1692077)
Supplement: Supplementary file 1 [file DataSheet1.docx]

Supplementary Material

1. **Ethical approach**

The study protocol was conducted in accordance with the guidelines from the International Conference on Harmonisation–Good Clinical Practice and Declaration of Helsinki. The study protocol and informed consent form were approved by the Ethics Committee of the Pomeranian Medical University in Szczecin (approval number: KB-0012/279/06/16; KB-0012/150/03/18). Detailed patient’s medical data were collected from the Electronic Medical Record System in the ICU, anonymized and are available upon reasonable request and with the hospital's consent.

# Supplementary Materials and Methods

**2.1. Key eligibility criteria**

Key inclusion criteria for cases with isolated candidemia (C)

Subjects must meet all of the following inclusion criteria to be included to C subgroup:

1. proven candidemia confirmed with 1^st^ positive blood culture result for Candida after admission to ICU and before antifungal therapy administration;
2. established SOFA and APACHE scoring ;
3. ICU stay > 7 days;
4. age: adult non-neutropenic patients >18 years old.

Key exclusion criteria for cases with isolated candidemia (C)

Subjects excluded from C subgroup if they have any of the following conditions:

1. proven bacteremia confirmed with positive blood culture result for bacteria;
2. proven candidemia with positive blood culture <72 hours before ICU admission and at ICU admission;
3. invasive fungal infections other than candidemia (pulmonary and disseminated aspergillosis and other systemic fungal breakthrough infections);
4. antifungal prophylaxis;
5. iatrogenic secondary immunodeficiency, in particular hematooncological conditions;
6. chronic liver diseases including alcohol addiction;
7. chronic inflammatory diseases- autoimmunity and IBD;
8. renal replacement therapy;
9. diabetes;
10. focal urinary tract infection.

Key inclusion criteria for cases with candidaemia with bacterial co-infection (BC)

Subjects must meet all of the following inclusion criteria to be included to BC subgroup

1. proven candidemia and bacteremia, confirmed by positive blood culture result 1st for bacteria and 2^nd^ for Candida, before antifungal therapy administration;
2. established SOFA and APACHE scoring;
3. ICU stay > 7 days;
4. age: adult, non-neutropenic patients >18 years old.

Key exclusion criteria for cases with candidemia with bacterial co-infection (BC)

Subjects excluded from BC subgroup if they have any of the following conditions:

1. proven candidemia with positive blood culture <72 hours before ICU admission and at ICU admission;
2. invasive fungal infections other than candidemia (pulmonary and disseminated aspergillosis and other systemic fungal breakthrough infections);
3. antifungal prophylaxis;
4. iatrogenic secondary immunodeficiency, in particular hematooncological conditions;
5. chronic liver diseases including alcohol addiction;
6. chronic inflammatory diseases - autoimmunity and IBD;
7. renal replacement therapy;
8. diabetes;
9. focal urinary tract infection.

Key inclusion criteria for non-septic controls (CON)

Subjects must meet all of the following inclusion criteria to be included to CON:

1. negative 2 weekly blood cultures for bacteria and fungi prior sample collection for proteomic analysis;
2. no biochemical indications for sepsis at the moment of sample collection for proteomic analysis;
3. established SOFA and APACHE scoring;
4. ICU stay > 7 days;
5. age: adult non-neutropenic patients >18 years old.

Key exclusion criteria for non-septic controls (CON)

Subjects excluded from CON if they have any of the following conditions:

1. proven candidemia confirmed with positive blood culture result for Candida before <72 hours before ICU admission, at ICU admission and during ICU stay;
2. proven bacteremia, confirmed with positive blood culture result for bacteria

before<72 hours before ICU admission, at ICU admission and during ICU stay;

1. biochemical parameters indicating suspicion of sepsis development;
2. invasive fungal infections other than candidaemia (pulmonary and disseminated aspergillosis and other systemic fungal breakthrough infections);
3. antifungal prophylaxis;
4. iatrogenic secondary immunodeficiency, in particular hematooncological conditions;
5. chronic liver diseases including alcohol addiction;
6. chronic inflammatory diseases- autoimmunity and IBD;
7. renal replacement therapy;
8. diabetes;
9. focal urinary tract

Blood-culturing was established in the standardized BACT/ALERT 3D microbial detection system (bioMérieux), whereas microbiological causes of sepsis were classified with high confidence by MALDI-Biotyper mass spectrometry (Bruker Daltonics).

**Supplementary Table 1**. **List of 92-inflammation-related proteins included** **in OLINK^®^ Target 96 Inflammation Panel.**

| **No** | **OlinkID** | **UniProt** | **Assay abbreviation** | **Other**  **abbreviation** | **Protein name** |
| --- | --- | --- | --- | --- | --- |
| 1. | OID00471 | P10145 | IL8 (CXCL8) | CXCL8 | Interleukin-8 |
| 2. | OID00472 | P15692 | VEGFA |  | Vascular endothelial growth factor A |
| 3. | OID05124 | P01732 | CD8A |  | T-cell surface glycoprotein CD8 alpha chain |
| 4. | OID00474 | P80098 | MCP-3 (CCL7) | CCL7 | Monocyte-chemotactic protein -3/ C-C motif chemokine 7 |
| 5. | OID00475 | P39905 | GDNF |  | Glial cell line-derived neurotrophic factor |
| 6. | OID00476 | Q9H5V8 | CDCP1 |  | CUB domain-containing protein 1 |
| 7. | OID00477 | Q9BZW8 | CD244 |  | Natural killer cell receptor 2B4 |
| 8. | OID00478 | P13232 | IL7 |  | Interleukin-7 |
| 9. | OID00479 | O00300 | OPG | TNFRSF11 | Osteoprotegerin /Tumor necrosis factor receptor superfamily member 11B |
| 10. | OID00480 | P01137 | LAP TGF-beta-1 | TGFB1 | Latency-associated peptide transforming growth factor beta-1 / transforming growth factor beta-1proprotein |
| 11. | OID00481 | P00749 | uPA | PLAU | Urokinase-type plasminogen activator |
| 12. | OID00482 | P05231 | IL6 |  | Interleukin -6 |
| 13. | OID00483 | Q9P0M4 | IL-17C |  | Interleukin-17C |
| 14. | OID00484 | P13500 | MCP-1 | CCL2 | Monocyte-chemotactic protein -1/C-C motif chemokine 2 |
| 15. | OID00485 | Q16552 | IL-17A |  | Interleukin-17A |
| 16. | OID00486 | O14625 | CXCL11 |  | C-X-C motif chemokine 11 |
| 17. | OID00487 | O15169 | AXIN1 |  | Axin-1 |
| 18. | OID00488 | P50591 | TRAIL | TNFSF10 | Tumor necrosis factor (TNF)-Related Apoptosis Inducing Ligand/Tumor necrosis factor ligand superfamily member 10 |
| 19. | OID00489 | Q9UHF4 | IL-20RA |  | Interleukin-20 receptor subunit alpha |
| 20. | OID00490 | Q07325 | CXCL9 |  | C-X-C motif chemokine 9 |
| 21. | OID00491 | P28325 | CST5 |  | Cystatin-D |
| 22. | OID00492 | P14784 | IL-2RB |  | Interleukin-2 receptor subunit beta |
| 23. | OID00493 | P01583 | IL-1 alpha | IL1A | Interleukin-1 alpha |
| 24. | OID00494 | P13725 | OSM |  | Oncostatin-M |
| 25. | OID00495 | P60568 | IL2 |  | Interleukin-2 |
| 26. | OID00496 | P09341 | CXCL1 |  | C-X-C motif chemokine 1/Growth-regulated alpha protein |
| 27. | OID00497 | Q969D9 | TSLP |  | Thymic stromal lymphopoietin |
| 28. | OID00498 | P13236 | CCL4 |  | C-C motif chemokine 4 |
| 29. | OID00499 | P30203 | CD6 |  | T-cell differentiation antigen CD6 |
| 30. | OID00500 | P21583 | SCF | KITLG | Stem-cell factor/Kit ligand |
| 31. | OID00501 | Q14116 | IL18 |  | Interleukin-18 |
| 32. | OID00502 | Q13291 | SLAMF1 |  | Signaling lymphocytic activation molecule |
| 33. | OID00503 | P01135 | TGF-alpha | TGFA | Protransforming growth factor alpha |
| 34. | OID00504 | Q99616 | MCP-4 | CCL13 | Monocyte-chemotactic protein-4/C-C motif chemokine 13 |
| 35. | OID00505 | P51671 | CCL11 |  | Eotaxin |
| 36. | OID00506 | O43557 | TNFSF14 |  | Tumor necrosis factor ligand superfamily member 14 |
| 37. | OID00507 | Q9GZV9 | FGF-23 |  | Fibroblast growth factor 23 |
| 38. | OID00508 | Q13651 | IL-10RA |  | Interleukin-10 receptor subunit alpha |
| 39. | OID00509 | P12034 | FGF-5 |  | Fibroblast growth factor 5 |
| 40. | OID00510 | P03956 | MMP-1 |  | Interstitial collagenase |
| 41. | OID00511 | P42702 | LIF-R |  | Leukemia inhibitory factor receptor |
| 42. | OID00512 | Q9NSA1 | FGF-21 |  | Fibroblast growth factor 21 |
| 43. | OID00513 | Q99731 | CCL19 |  | C-C motif chemokine 19 |
| 44. | OID00514 | Q13261 | IL-15RA |  | Interleukin-15 receptor subunit alpha |
| 45. | OID00515 | Q08334 | IL-10RB |  | Interleukin-10 receptor subunit beta |
| 46. | OID00516 | Q8N6P7 | IL-22 RA1 |  | Interleukin-22 receptor subunit alpha-1 |
| 47. | OID00517 | Q13478 | IL-18R1 |  | Interleukin-18 receptor 1 |
| 48. | OID00518 | Q9NZQ7 | PD-L1 | CD274 | Programmed cell death 1 ligand 1 |
| 49. | OID00519 | P01138 | Beta-NGF | NGF | Beta-nerve growth factor |
| 50. | OID00520 | P42830 | CXCL5 |  | C-X-C motif chemokine 5 |
| 51. | OID00521 | O14788 | TRANCE | TNFSF11 | TNF-related activation-induced cytokine /Tumor necrosis factor ligand superfamily member 11 |
| 52. | OID00522 | P14210 | HGF |  | Hepatocyte growth factor |
| 53. | OID00523 | P29460 | IL-12B |  | Interleukin-12 subunit beta |
| 54. | OID00524 | Q13007 | IL-24 |  | Interleukin-24 |
| 55. | OID00525 | P35225 | IL13 |  | Interleukin-13 |
| 56. | OID00526 | Q5T4W7 | ARTN |  | Artemin |
| 57. | OID00527 | P09238 | MMP-10 |  | Stromelysin-2 |
| 58. | OID00528 | P22301 | IL10 |  | Interleukin-10 |
| 59. | OID05548 | P01375 | TNF |  | Tumor nerosis factor |
| 60. | OID00530 | P55773 | CCL23 |  | C-C motif chemokine 23 |
| 61. | OID00531 | P06127 | CD5 |  | T-cell surface glycoprotein CD5 |
| 62. | OID00532 | P10147 | CCL3 |  | C-C motif chemokine 3 |
| 63. | OID00533 | P49771 | Flt3L | FLT3LG | Fms-related tyrosine kinase 3 ligand |
| 64. | OID00534 | P80162 | CXCL6 |  | C-X-C motif chemokine 6 |
| 65. | OID00535 | P02778 | CXCL10 |  | C-X-C motif chemokine 10 |
| 66. | OID00536 | Q13541 | 4E-BP1 | EIF4EBP1 | Eukaryotic translation initiation factor 4E-binding protein 1 |
| 67. | OID00537 | Q9NYY1 | IL-20 |  | Interleukin-20 |
| 68. | OID00538 | Q8IXJ6 | SIRT2 |  | NAD-dependent protein deacetylase sirtuin-2 |
| 69. | OID00539 | Q9NRJ3 | CCL28 |  | C-C motif chemokine 28 |
| 70. | OID01213 | Q8NFT8 | DNER |  | Delta and Notch-like epidermal growth factor-related receptor |
| 71. | OID00541 | P80511 | EN-RAGE | S100A12 | Protein S100-A12 |
| 72. | OID00542 | P25942 | CD40 |  | Tumor necrosis factor receptor superfamily member 5 |
| 73. | OID00543 | O95760 | IL33 |  | Interleukin-33 |
| 74. | OID05547 | P01579 | IFN-gamma | IFNG | Interferon gamma |
| 75. | OID00545 | O95750 | FGF-19 |  | Fibroblast growth factor 19 |
| 76. | OID00546 | P05112 | IL4 |  | Interleukin-4 |
| 77. | OID00547 | P15018 | LIF |  | Leukemia inhibitory factor |
| 78. | OID00548 | Q99748 | NRTN |  | Neurturin |
| 79. | OID00549 | P80075 | MCP-2 | CCL8 | Monocyte-chemotactic protein -2/C-C motif chemokine 8 |
| 80. | OID00550 | Q14790 | CASP-8 |  | Caspase-8 |
| 81. | OID00551 | O15444 | CCL25 |  | C-C motif chemokine 25 |
| 82. | OID00552 | P78423 | CX3CL1 |  | Fractalkine |
| 83. | OID00553 | Q07011 | TNFRSF9 |  | Tumor necrosis factor receptor superfamily member 9 |
| 84. | OID00554 | P20783 | NT-3 | NTF3 | Neurotrophin-3 |
| 85. | OID00555 | O43508 | TWEAK | TNFSF12 | Tumor necrosis factor ligand superfamily member 12 |
| 86. | OID00556 | P78556 | CCL20 |  | C-C motif chemokine 20 |
| 87. | OID00557 | P50225 | ST1A1 | SULT1A1 | Sulfotransferase 1A1 |
| 88. | OID00558 | O95630 | STAMBP |  | STAM-binding protein |
| 89. | OID00559 | P05113 | IL5 |  | Interleukin-5 |
| 90. | OID00560 | P00813 | ADA |  | Adenosine deaminase |
| 91. | OID00561 | P01374 | TNFB | LTA | Lymphotoxin-alpha |
| 92. | OID00562 | P09603 | CSF-1 |  | Macrophage colony-stimulating factor 1 |

92 –inflammation-related mediators and controls of assay performance and each sample quality
 were included in n OLINK^®^ Target 96 Inflammation Panel. Number of samples, that passed the quality
 control was 94%

**2.2. Quality control**

Protein expression was reported as log2 transformation in a normalized protein expression (NPX) scale. Four internal controls were added to each sample to monitor the quality of the assay performance, as well as the quality of the individual samples. Protein expression detected in at least 75% of the samples and samples that deviated less than 0.3 NPX from the median were included as they passed the quality control (QC) and the required threshold for complete observations. Samples that failed QC were excluded from differential expression (DE) analysis. Inflammatory proteins with expression below the low limit of detection (LLOD) were excluded from the DE analysis of detected proteins between groups.

**2.3. Statistics**

The protein expression data were first normalised to account for variability in measurement scales. The dataset comprised Normalised Protein eXpression (NPX) values for 92 cytokines, presented on a log2 scale, for different patient groups. Due to the distinct NPX value ranges for each protein, comparisons between different proteins were not performed. To reduce dimensionality and visualise data variability between the groups, we performed principal component analysis (PCA) based on NPX data. The scikit-learn package was used for PCA, and the results were visualised with convex hulls using the matplotlib and NumPy libraries. The first two principal components were plotted, representing the maximum variance in the data. Then, hierarchical clustering was performed to identify patterns and clusters within protein expression data. The seaborn package created a heatmap with dendrograms, providing a visual representation of clustering based on Ward’s method and Euclidean distance.

To identify inflammatory proteomes, differential expression analysis between the groups (C and BC and CON) were conducted using the SciPy package. Numerical data for each group were selected, and group means were calculated. Fold changes were computed as the log2 ratio of the means between the groups. Pairwise comparisons among groups (CON, BC, and C) were assessed using Welch's t-test, suitable for unequal variances to identify differentially expressed proteins (DEPs). Volcano plots were generated to visualise DEPs. These plots, highlighting significant changes, displayed the log2 fold change against the -log10 p-value. The matplotlib and seaborn libraries were utilised for these visualisations. Multiple testing corrections among groups (CON and BC and C) were applied using the Benjamini-Hochberg false discovery rate (FDR) correction. This analysis was conducted using the statsmodels package. All statistical analyses and dataset visualisations were conducted using Python, leveraging packages such as Pandas, NumPy, SciPy, scikit-learn, statsmodels, matplotlib, and seaborn. A p-value < 0.05 was considered statistically significant for all tests.

Continuous variables were expressed as means or medians, depending on their distribution. Categorical variables (sex, ICU mortality, DCD, sepsis in ICU, abdominal surgery, total parenteral nutrition, multifocal Candida colonisation, candidemia aetiology, azole-resistant mediated candidemia) were expressed as numbers and percentages and were compared using the Chi-squared test. Continuous variables (age, SOFA, APACHE II, Candida score, CRP, PCT) that were not normally distributed were compared using the Mann-Whitney U test.

1. **Results**

**Supplementary Table 2.**

**Differential expression of inflammation-related proteins in candidemia cases versus non-septic ICU controls**. Table displays statistical p-value for 75 circulating proteins detected by the OLINK Inflammatory Panel with n = 24 patients with candidemia (C+BC) over n = 20 non-septic control subjects included. The 52 of 75 detected proteins were statistically differentially expressed proteins (DEPs; p-value < 0.05) between cases and controls and highlighted as statistical (true) significant by numbers from 1 to 52 (bold font). Analysis based on NPX (Normalized Protein eXpression).

| **Number** | **Feature /Protein** | **Fold**  **Change** | **raw**  **p-value** | **Significant  cases vs. control** |
| --- | --- | --- | --- | --- |
| **1.** | **CXCL11** | 0.4709870330150043 | 2.688619362892108e-09 | True |
| **2.** | **LAP TGF-beta-1** | 0.23687525304865484 | 2.6400332885841134e-07 | True |
| **3.** | **CD40** | 0.1526713588627272 | 7.560266824607207e-07 | True |
| **4.** | **CXCL1** | 0.26052475204396375 | 8.243020295161354e-07 | True |
| **5.** | **CXCL6** | 0.2227105798561471 | 1.6345591071132601e-06 | True |
| **6.** | **IL18** | 0.21932850645167307 | 2.1144924987610874e-06 | True |
| **7.** | **CXCL10** | 0.351783944184661 | 2.2729509468894018e-06 | True |
| **8.** | **uPA** | 0.1322174232283972 | 3.679025523557074e-06 | True |
| **9.** | **IL-18R1** | 0.16584902064103418 | 7.030770277266255e-06 | True |
| **10.** | **MCP-2** | 0.2516040097220514 | 9.510333499278666e-06 | True |
| **11.** | **VEGFA** | 0.10820920051527472 | 5.861817159427394e-05 | True |
| **12.** | **PD-L1** | 0.2555058267295479 | 5.289999867510807e-05 | True |
| **13.** | **TNF** | 0.6154912320921219 | 2.5091059241223734e-05 | True |
| **14.** | **IFN-gamma** | 0.7475188217295762 | 1.1882441145947975e-05 | True |
| **15.** | **CX3CL1** | 0.4196114320691717 | 4.187484822440219e-05 | True |
| **16.** | **CXCL9** | 0.30167791702561575 | 0.00012472363025538906 | True |
| **17.** | **GDNF** | 0.8692640138088594 | 0.00013575071717335547 | True |
| **18.** | **CD5** | 0.2281616772004311 | 0.00035713793272827785 | True |
| **19.** | **EN-RAGE** | 0.2061904698518114 | 0.00039344839565291003 | True |
| **20.** | **CCL3** | 0.27644774060886673 | 0.00039400284486201245 | True |
| **21.** | **TNFB** | 0.5087796990752601 | 0.0004908263934663077 | True |
| **22.** | **CD244** | 0.12453879222402262 | 0.0006846752380528076 | True |
| **23.** | **MCP-4** | 0.1297415119019703 | 0.0008218949546255406 | True |
| **24.** | **Flt3L** | 0.19265903415194233 | 0.0008645886274408899 | True |
| **25.** | **CDCP1** | 0.34329289402783053 | 0.00093062393949137 | True |
| **26.** | **IL8** | 0.314403825530491 | 0.0010744345369785137 | True |
| **27.** | **IL-15RA** | 0.8320048928676583 | 0.0010840560589840272 | True |
| **28.** | **IL-17A** | 0.913411606443515 | 0.0011295653084864282 | True |
| **29.** | **TNFSF14** | 0.19731471715111246 | 0.0012513983384389185 | True |
| **30.** | **OPG** | 0.08811652058091464 | 0.002033980787933766 | True |
| **31.** | **CCL28** | 0.4724725602929472 | 0.002067607441228326 | True |
| **32.** | **MCP-3** | 0.43902429661018466 | 0.002190893434403032 | True |
| **33.** | **CCL19** | 0.18719102406627708 | 0.0023783523077702266 | True |
| **34.** | **STAMBP** | 0.2781963379221876 | 0.002794591971664971 | True |
| **35.** | **IL-12B** | 0.44007304107417816 | 0.00288766303454055 | True |
| **36.** | **TRAIL** | 0.11345417201040746 | 0.0032705302815590398 | True |
| **37.** | **CASP-8** | 0.21920666241255293 | 0.00447574302241073 | True |
| **38.** | **LIF-R** | 0.13778618635060064 | 0.0057503856833283455 | True |
| **39.** | **CCL11** | 0.14021709454296025 | 0.006006235873194063 | True |
| **40.** | **SLAMF1** | 0.3735514402719242 | 0.007746270621510799 | True |
| **41.** | **IL7** | 0.4079373124695565 | 0.00998416004418956 | True |
| **42.** | **IL-10RB** | 0.11584870277443579 | 0.010815740567212717 | True |
| **43.** | **AXIN1** | 0.4264696069863296 | 0.011013156658352728 | True |
| **44.** | **CXCL5** | 0.11033701507374576 | 0.011508926720299028 | True |
| **45.** | **TNFRSF9** | 0.24475215424886104 | 0.012971297413761964 | True |
| **46.** | **SIRT2** | 0.28368891011384045 | 0.013345144855001888 | True |
| **47.** | **MCP-1** | 0.09599087713800453 | 0.020870367861647768 | True |
| **48.** | **HGF** | 0.07736468382849958 | 0.024132470475897876 | True |
| **49.** | **SCF** | -0.1231933034466448 | 0.02627024555667773 | True |
| **50.** | **CCL4** | 0.13673472309311957 | 0.034316481061185664 | True |
| **51.** | **CD8A** | 0.15797821842535165 | 0.03615638459631472 | True |
| **52.** | **CCL20** | 0.15995667680952708 | 0.038308001344702226 | True |
| 53. | IL6 | 0.10486436634354772 | 0.4028662886748735 | False |
| 54. | IL-17C | -0.2839265832025381 | 0.18852456044514745 | False |
| 55. | CST5 | -0.013909083029839134 | 0.7527542560707918 | False |
| 56. | OSM | 0.11608045080049802 | 0.13397889438398672 | False |
| 57. | CD6 | 0.08342688682787056 | 0.15956657066401056 | False |
| 58. | TGF-alpha | 0.17541474021165698 | 0.05164110628998679 | False |
| 59. | FGF-23 | 0.29017496250878844 | 0.4188233070740205 | False |
| 60. | IL-10RA | -0.24740261148036158 | 0.5610769891086255 | False |
| 61. | FGF-5 | -0.27459227890633375 | 0.06636783788887904 | False |
| 62. | MMP-1 | 0.04698930850942754 | 0.06380521298466542 | False |
| 63. | FGF-21 | -0.05469925166970779 | 0.6811018282294642 | False |
| 64. | TRANCE | 0.23274097010249678 | 0.05146293581886102 | False |
| 65. | MMP-10 | 0.061547351428565045 | 0.1810714326308171 | False |
| 66. | IL10 | 0.3883942195862962 | 0.07124865972998759 | False |
| 67. | CCL23 | 0.04579988401177802 | 0.16115687041245846 | False |
| 68. | 4E-BP1 | 0.1878881234327829 | 0.12429764008703412 | False |
| 69. | DNER | -0.07665461839856312 | 0.08992972475975211 | False |
| 70. | FGF-19 | -0.029323971383633424 | 0.7694089604766594 | False |
| 71. | CCL25 | 0.02122548150174803 | 0.7175493704760457 | False |
| 72. | TWEAK | 0.07841613204633614 | 0.11781910526292036 | False |
| 73. | ST1A1 | 0.0865040814706467 | 0.10179107533955395 | False |
| 74. | ADA | -0.041367548650025246 | 0.651683731584166 | False |
| 75. | CSF-1 | 0.027037765893056228 | 0.09801864020025249 | False |

The following 17 inflammation-related proteins were below low limit of detection (LLOD) specific for each protein with missing data from 75% to 99%: IL-20 RA, IL-2RB, IL-1, IL-2, TSLP, IL-22RA1, beta-NGF, IL-24, IL-13, ARTN, IL-20, IL-33, IL-4, NRTN, NT-3, IL5, LIF.

**Supplementary Table 3.**

**Significantly up- and down-regulated proteins in candidemia cases versus non-septic ICU controls.** Rows 1 to 51 display 51 upregulated, differentially expressed proteins (DEPs) whereas row 52 displays 1 down-regulated DEPs detected by the OLINK Inflammatory Panel with n = 24 patients with candidemia (C+BC) over n = 20 non-septic control subjects included. Analysis based on NPX (Normalized Protein eXpression).

| **Number** | **Feature /Protein** | **Fold Change** | **raw p-value** |
| --- | --- | --- | --- |
| **UP-REGULATED protein expression**  cases versus control | | | |
| 1. | CXCL11 | 0.4709870330150043 | 2.688619362892108e-09 |
| 2. | LAP TGF-beta-1 | 0.23687525304865484 | 2.6400332885841134e-07 |
| 3. | CD40 | 0.1526713588627272 | 7.560266824607207e-07 |
| 4. | CXCL1 | 0.26052475204396375 | 8.243020295161354e-07 |
| 5. | CXCL6 | 0.2227105798561471 | 1.6345591071132601e-06 |
| 6. | IL18 | 0.21932850645167307 | 2.1144924987610874e-06 |
| 7. | CXCL10 | 0.351783944184661 | 2.2729509468894018e-06 |
| 8. | uPA | 0.1322174232283972 | 3.679025523557074e-06 |
| 9. | IL-18R1 | 0.16584902064103418 | 7.030770277266255e-06 |
| 10. | MCP-2 | 0.2516040097220514 | 9.510333499278666e-06 |
| 11. | VEGFA | 0.10820920051527472 | 5.861817159427394e-05 |
| 12. | PD-L1 | 0.2555058267295479 | 5.289999867510807e-05 |
| 13. | TNF | 0.6154912320921219 | 2.5091059241223734e-05 |
| 14. | IFN-gamma | 0.7475188217295762 | 1.1882441145947975e-05 |
| 15. | CX3CL1 | 0.4196114320691717 | 4.187484822440219e-05 |
| 16. | CXCL9 | 0.30167791702561575 | 0.00012472363025538906 |
| 17. | GDNF | 0.8692640138088594 | 0.00013575071717335547 |
| 18. | CD5 | 0.2281616772004311 | 0.00035713793272827785 |
| 19. | EN-RAGE | 0.2061904698518114 | 0.00039344839565291003 |
| 20. | CCL3 | 0.27644774060886673 | 0.00039400284486201245 |
| 21. | TNFB | 0.5087796990752601 | 0.0004908263934663077 |
| 22. | CD244 | 0.12453879222402262 | 0.0006846752380528076 |
| 23. | MCP-4 | 0.1297415119019703 | 0.0008218949546255406 |
| 24. | Flt3L | 0.19265903415194233 | 0.0008645886274408899 |
| 25. | CDCP1 | 0.34329289402783053 | 0.00093062393949137 |
| 26. | IL8 | 0.314403825530491 | 0.0010744345369785137 |
| 27. | IL-15RA | 0.8320048928676583 | 0.0010840560589840272 |
| 28. | IL-17A | 0.913411606443515 | 0.0011295653084864282 |
| 29. | TNFSF14 | 0.19731471715111246 | 0.0012513983384389185 |
| 30. | OPG | 0.08811652058091464 | 0.002033980787933766 |
| 31. | CCL28 | 0.4724725602929472 | 0.002067607441228326 |
| 32. | MCP-3 | 0.43902429661018466 | 0.002190893434403032 |
| 33. | CCL19 | 0.18719102406627708 | 0.0023783523077702266 |
| 34. | STAMBP | 0.2781963379221876 | 0.002794591971664971 |
| 35. | IL-12B | 0.44007304107417816 | 0.00288766303454055 |
| 36. | TRAIL | 0.11345417201040746 | 0.0032705302815590398 |
| 37. | CASP-8 | 0.21920666241255293 | 0.00447574302241073 |
| 38. | LIF-R | 0.13778618635060064 | 0.0057503856833283455 |
| 39. | CCL11 | 0.14021709454296025 | 0.006006235873194063 |
| 40. | SLAMF1 | 0.3735514402719242 | 0.007746270621510799 |
| 41. | IL7 | 0.4079373124695565 | 0.00998416004418956 |
| 42. | IL-10RB | 0.11584870277443579 | 0.010815740567212717 |
| 43. | AXIN1 | 0.4264696069863296 | 0.011013156658352728 |
| 44. | CXCL5 | 0.11033701507374576 | 0.011508926720299028 |
| 45. | TNFRSF9 | 0.24475215424886104 | 0.012971297413761964 |
| 46. | SIRT2 | 0.28368891011384045 | 0.013345144855001888 |
| 47. | MCP-1 | 0.09599087713800453 | 0.020870367861647768 |
| 48. | HGF | 0.07736468382849958 | 0.024132470475897876 |
| 49. | CCL4 | 0.13673472309311957 | 0.034316481061185664 |
| 50. | CD8A | 0.15797821842535165 | 0.03615638459631472 |
| 51. | CCL20 | 0.15995667680952708 | 0.038308001344702226 |
| **DOWN-REGULATED protein expression**  cases versus control | | | |
| 52. | SCF | -0.1231933034466448 | 0.02627024555667773 |

**Supplementary Table 4.**

**Differential expression of inflammation–related proteins in isolated candidemia versus non-septic ICU controls.** Rows 1 to 48 display differentially expressed proteins (DEPs), highlighted as statistical (true) significant, detected by the OLINK Inflammatory Panel in n = 12 patients with isolated candidemia (C) compared to n = 20 non-septic control subjects. These include 46 up- and 2 downregulated proteins (IL-17C and SCF, rows 35 and 44 respectively, bold italic font). Analysis based on NPX (Normalized Protein eXpression).

| **Number** | **Feature/ Protein** | **Fold Change** | **Raw p-value** | **Significant**  **C vs CON** |
| --- | --- | --- | --- | --- |
| 1. | CXCL6 | 0.25808330057852646 | 1.4915281357953666e-06 | True |
| 2. | LAP TGF-beta-1 | 0.3036234295110762 | 5.769952093251432e-06 | True |
| 3. | CXCL1 | 0.26085820767016965 | 1.8409121872831952e-05 | True |
| 4. | CXCL11 | 0.45597031082659445 | 3.453954735666117e-05 | True |
| 5. | CD5 | 0.29510916067164045 | 6.279864005619314e-05 | True |
| 6. | EN-RAGE | 0.23391448396853096 | 0.00019458246168165217 | True |
| 7. | IFN-gamma | 0.6186115998714158 | 0.0002309317471128096 | True |
| 8. | CD244 | 0.1697525236255663 | 0.0003371644505320124 | True |
| 9. | MCP-2 | 0.21555556118682148 | 0.0004909672672838113 | True |
| 10. | CXCL10 | 0.31316814244378305 | 0.0006112644204259214 | True |
| 11. | IL-18R1 | 0.15615773370787173 | 0.0006423028966731367 | True |
| 12. | uPA | 0.14436809151572205 | 0.0008813845689766264 | True |
| 13. | CD40 | 0.1513925590310038 | 0.0010641845174225797 | True |
| 14. | TRANCE | 0.41067107219887194 | 0.001084018481315796 | True |
| 15. | TNFSF14 | 0.24851801836222295 | 0.0012554774450921871 | True |
| 16. | VEGFA | 0.11419266606208602 | 0.001425455298646823 | True |
| 17. | IL18 | 0.23126785046865347 | 0.0015490948482192008 | True |
| 18. | TNFB | 0.5024888856268288 | 0.0018547552256110516 | True |
| 19. | PD-L1 | 0.24287670959120325 | 0.0019316114094675525 | True |
| 20. | CXCL9 | 0.2864689156495028 | 0.0019758764816266176 | True |
| 21. | TRAIL | 0.1360822210951501 | 0.002567425433495197 | True |
| 22. | GDNF | 0.8660775091202949 | 0.0034311269446835398 | True |
| 23. | CCL11 | 0.14972695915113582 | 0.0035299454368690297 | True |
| 24. | CXCL5 | 0.13570798016076685 | 0.003666728025376434 | True |
| 25. | CDCP1 | 0.4091878797858862 | 0.0036669286533308693 | True |
| 26. | Flt3L | 0.1986931919925481 | 0.0040103692451037105 | True |
| 27. | AXIN1 | 0.5496135149346902 | 0.004587672534345344 | True |
| 28. | CCL28 | 0.5817420276757993 | 0.004716366949662476 | True |
| 29. | LIF-R | 0.16954083608021583 | 0.0050036432826140705 | True |
| 30. | TNF | 0.5751041703463008 | 0.005332431099870044 | True |
| 31. | MCP-4 | 0.12398598207398354 | 0.00668125846600937 | True |
| 32. | IL-12B | 0.46272919699548654 | 0.008094119442241513 | True |
| 33. | CX3CL1 | 0.29932515164130413 | 0.008300941913744333 | True |
| 34. | CASP-8 | 0.2718863168749197 | 0.010984007574859097 | True |
| 35. | ***IL-17C*** | ***-0.6683955663949019*** | ***0.012601552141009205*** | ***True*** |
| 36. | IL-17A | 0.9150693927290794 | 0.01632642743649851 | True |
| 37. | STAMBP | 0.28086993780406266 | 0.022053784938693824 | True |
| 38. | CD8A | 0.1895877181125437 | 0.02344778955391016 | True |
| 39. | CD6 | 0.17069544793153973 | 0.02417021046298331 | True |
| 40. | CCL3 | 0.22992822263704513 | 0.02524956087118955 | True |
| 41. | OPG | 0.0701370181423815 | 0.0255137275332246 | True |
| 42. | SLAMF1 | 0.4286192338964795 | 0.030965459752470725 | True |
| 43. | TWEAK | 0.12966448924613586 | 0.03163120862623765 | True |
| 44. | ***SCF*** | ***-0.18355667817663482*** | ***0.03352791269417203*** | ***True*** |
| 45. | IL8 | 0.2571657487465437 | 0.041399944082351176 | True |
| 46. | IL-15RA | 0.7456203729223719 | 0.043561575214162505 | True |
| 47. | MMP-1 | 0.05252568097629027 | 0.04444791266299207 | True |
| 48. | SIRT2 | 0.3017915078755116 | 0.04510224326558265 | True |
| 49. | CCL19 | 0.1479949739433488 | 0.05639029097545059 | False |
| 50. | IL7 | 0.40631184467087006 | 0.05687961562743511 | False |
| 51. | TGF-alpha | 0.17949042983429264 | 0.08001598184517486 | False |
| 52. | TNFRSF9 | 0.22681002759750227 | 0.08636819632733646 | False |
| 53. | FGF-5 | -0.28462072405047745 | 0.12333347298488453 | False |
| 54. | MCP-3 | 0.37327543990696616 | 0.12604611083357095 | False |
| 55. | ST1A1 | 0.08039799720595465 | 0.1548552780146 | False |
| 56. | MCP-1 | 0.0670464709330792 | 0.1624412207396758 | False |
| 57. | 4E-BP1 | 0.16498388441849404 | 0.17903564999488286 | False |
| 58. | IL-10RB | 0.09470703294119652 | 0.19530117755756196 | False |
| 59. | CCL20 | 0.1242001942869265 | 0.20585895245273247 | False |
| 60. | DNER | -0.09625258137310794 | 0.23719739499484072 | False |
| 61. | CCL4 | 0.09896934279762583 | 0.24916337653539353 | False |
| 62. | CSF-1 | 0.02063441253800521 | 0.260736017402487 | False |
| 63. | OSM | 0.11962101923786891 | 0.2746419977815902 | False |
| 64. | HGF | 0.05175354512362423 | 0.282124361683862 | False |
| 65. | MMP-10 | 0.06246711648917006 | 0.2922286202387112 | False |
| 66. | IL-10RA | -0.5230169546265028 | 0.35380956503777683 | False |
| 67. | FGF-21 | -0.15015917784393054 | 0.3792631227079629 | False |
| 68. | FGF-19 | -0.07655862297710814 | 0.5195098221410479 | False |
| 69. | CCL23 | 0.022977132458444145 | 0.5449865892903201 | False |
| 70. | IL10 | 0.16545064087271935 | 0.5476608332039612 | False |
| 71. | CST5 | -0.026409600442569722 | 0.6467975373690658 | False |
| 72. | ADA | -0.04723845428159514 | 0.6925465980363306 | False |
| 73. | CCL25 | 0.02372403670013426 | 0.7206494548542204 | False |
| 74. | FGF-23 | -0.0665480673658875 | 0.8819158152382913 | False |
| 75. | IL6 | -0.012636951558631416 | 0.9320600668728456 | False |

**Supplementary Table 5. Differential expression of inflammation–related proteins in candidemia with bacterial co-infection versus non-septic ICU controls.** Rows 1 to 44 display 44 upregulated, differentially expressed proteins (DEPs), highlighted as statistical (true) significant, detected by the OLINK Inflammatory Panel with n = 12 patients with candidemia with bacterial co-infection (BC) over n = 20 non-septic control subjects included. Analysis based on NPX (Normalized Protein eXpression).

| **Number** | **Feature /Protein** | **Fold Change** | **Raw p-value** | **Significant**  **BC v s CON** |
| --- | --- | --- | --- | --- |
| 1. | CXCL11 | 0.48461638686208486 | 1.329377948540417e-06 | True |
| 2. | CD40 | 0.1538425971055888 | 8.467204366773528e-06 | True |
| 3. | uPA | 0.12098869057444057 | 9.443434439930739e-06 | True |
| 4. | CXCL1 | 0.2602190166654453 | 1.895323625599366e-05 | True |
| 5. | CXCL10 | 0.38629611266601155 | 7.66539331724147e-05 | True |
| 6. | IL18 | 0.2082966246419913 | 9.714374858068107e-05 | True |
| 7. | VEGFA | 0.10270247121372544 | 0.00010807444194985354 | True |
| 8. | CXCL6 | 0.1895056040128437 | 0.0001296212871092168 | True |
| 9. | TNF | 0.651545035032536 | 0.00022713060095507436 | True |
| 10. | CX3CL1 | 0.5217033706437003 | 0.00023102130593168773 | True |
| 11. | MCP-3 | 0.4967701013694543 | 0.0002463101374615157 | True |
| 12. | IL-18R1 | 0.17467587117969086 | 0.00035959203723340304 | True |
| 13. | IFN-gamma | 0.8563484679670613 | 0.00045151151248549944 | True |
| 14. | MCP-2 | 0.2838753577307872 | 0.0006085163698723915 | True |
| 15. | LAP TGF-beta-1 | 0.17285211051142424 | 0.0007024742935519817 | True |
| 16. | CCL3 | 0.31781176569900704 | 0.0007517706860179959 | True |
| 17. | PD-L1 | 0.2669861953294577 | 0.0009523441346031923 | True |
| 18. | IL-15RA | 0.9068890813201973 | 0.0017028649123621976 | True |
| 19. | CCL19 | 0.2222086076986256 | 0.0021203098399035945 | True |
| 20. | IL8 | 0.3649490054727238 | 0.002527364907436558 | True |
| 21. | IL-10RB | 0.1349601133884313 | 0.0033460828310171394 | True |
| 22. | CXCL9 | 0.3154800397235343 | 0.003880023223449365 | True |
| 23. | Flt3L | 0.18710546376435694 | 0.004691788998270376 | True |
| 24. | MCP-1 | 0.12202259000308338 | 0.005207588677278293 | True |
| 25. | GDNF | 0.8721788065451427 | 0.005277970443211109 | True |
| 26. | OPG | 0.10440318098718168 | 0.005713973301011355 | True |
| 27. | MCP-4 | 0.13499731909050805 | 0.008823595368332333 | True |
| 28. | EN-RAGE | 0.18030010484170572 | 0.009069496609385282 | True |
| 29. | CDCP1 | 0.2801255399326513 | 0.009215544839192059 | True |
| 30. | TNFB | 0.5145222796393975 | 0.010671885626930451 | True |
| 31. | TNFRSF9 | 0.2610053568608941 | 0.010893876872773588 | True |
| 32. | STAMBP | 0.27574117782241797 | 0.011055606016519377 | True |
| 33. | HGF | 0.10044873565477284 | 0.012330584059663835 | True |
| 34. | IL-17A | 0.9118902937551767 | 0.016409351686439852 | True |
| 35. | CCL28 | 0.3644748272257675 | 0.027357421443186654 | True |
| 36. | SLAMF1 | 0.32115686661998427 | 0.027615766339358624 | True |
| 37. | TNFSF14 | 0.14872640598265774 | 0.02848237853022306 | True |
| 38. | CD5 | 0.16393847468797074 | 0.029656354509294693 | True |
| 39. | CCL4 | 0.17050545922753826 | 0.039467109775439595 | True |
| 40. | IL10 | 0.5663151905553758 | 0.04096408028122893 | True |
| 41. | IL7 | 0.40942571749804496 | 0.04267085387511951 | True |
| 42. | IL-12B | 0.418987632402109 | 0.0445940440747616 | True |
| 43. | CCL11 | 0.13144430514206148 | 0.04754709090714921 | True |
| 44. | TRAIL | 0.092395320563929 | 0.04775803169649845 | True |
| 45. | CSF-1 | 0.03288264353804299 | 0.05201181606606706 | False |
| 46. | SIRT2 | 0.2668929243715668 | 0.05612763729056208 | False |
| 47. | LIF-R | 0.10805070092121036 | 0.06744084175730182 | False |
| 48. | CCL20 | 0.1919727195347795 | 0.0712292385019592 | False |
| 49. | CASP-8 | 0.1691666317388036 | 0.07406435781503007 | False |
| 50. | CCL23 | 0.0664082351492126 | 0.07485774617946878 | False |
| 51. | CD244 | 0.08181002590516041 | 0.07825707022897298 | False |
| 52. | CXCL5 | 0.08668172133180821 | 0.09332557970781441 | False |
| 53. | DNER | -0.058920729903947436 | 0.10203191937481178 | False |
| 54. | TGF-alpha | 0.17166854956299626 | 0.1106829291567511 | False |
| 55. | MMP-1 | 0.04189556853214651 | 0.1507679416748008 | False |
| 56. | FGF-5 | -0.2654603762790254 | 0.15119989919709823 | False |
| 57. | MMP-10 | 0.060703718027288855 | 0.17264211455638925 | False |
| 58. | FGF-23 | 0.5541808129958167 | 0.17526174000027334 | False |
| 59. | CD8A | 0.12838156712415422 | 0.17851285149678198 | False |
| 60. | IL6 | 0.20476432075897072 | 0.18158322434972848 | False |
| 61. | OSM | 0.11282727892252616 | 0.1852208066050383 | False |
| 62. | AXIN1 | 0.3035382004072649 | 0.19157001500567866 | False |
| 63. | 4E-BP1 | 0.2085689523675647 | 0.21293215038000474 | False |
| 64. | SCF | -0.06999481878389838 | 0.2160906281884816 | False |
| 65. | ST1A1 | 0.09207871295786808 | 0.2202807447318194 | False |
| 66. | TWEAK | 0.029783560239318444 | 0.6325721607001152 | False |
| 67. | TRANCE | 0.04778947620017006 | 0.7391049107209101 | False |
| 68. | ADA | -0.03600679246915097 | 0.7803454993289827 | False |
| 69. | CCL25 | 0.018931331714585515 | 0.7811318875419633 | False |
| 70. | FGF-21 | 0.02758187004876136 | 0.8633772251550033 | False |
| 71. | FGF-19 | 0.012656567249846382 | 0.9117631763515526 | False |
| 72. | IL-10RA | -0.034012100986911604 | 0.9381446230825289 | False |
| 73. | CST5 | -0.002544653712728383 | 0.9556885557859061 | False |
| 74. | CD6 | -0.0014890339117387273 | 0.9834423233358214 | False |
| 75. | IL-17C | -0.0036304228252377387 | 0.9859036909979275 | False |

**Supplementary Fig. 1(A)** (C versus CON)
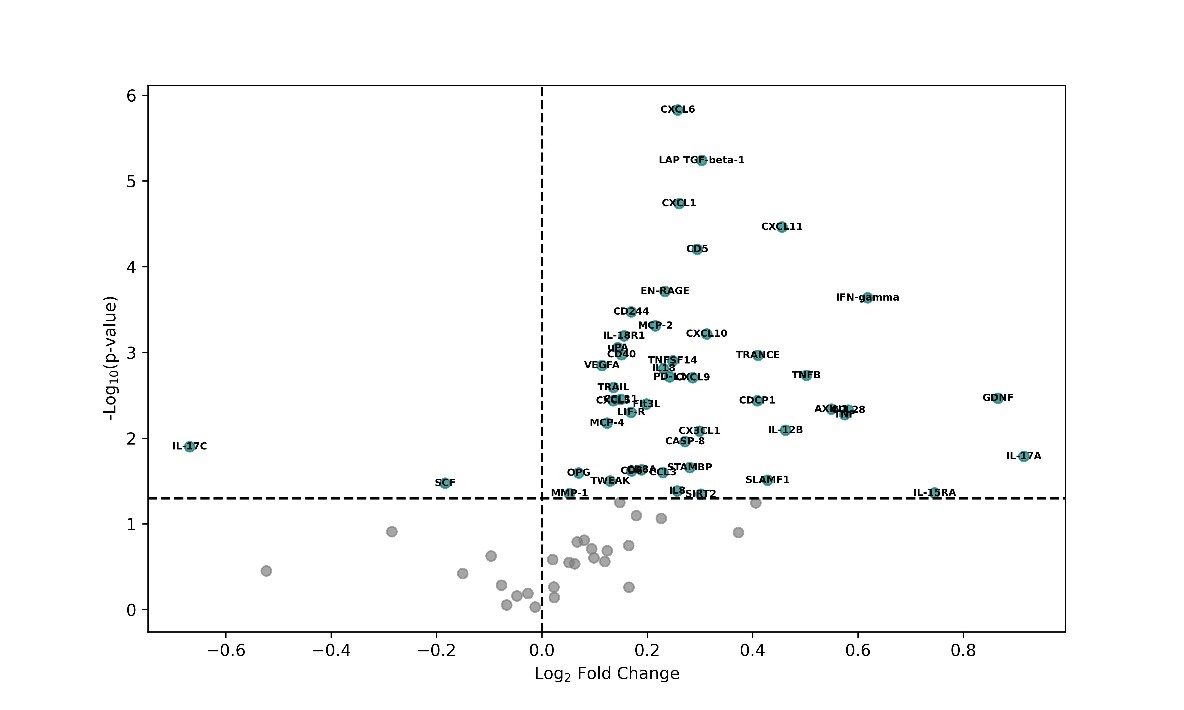


**Supplementary Fig. 1(B) (**BC versus CON)


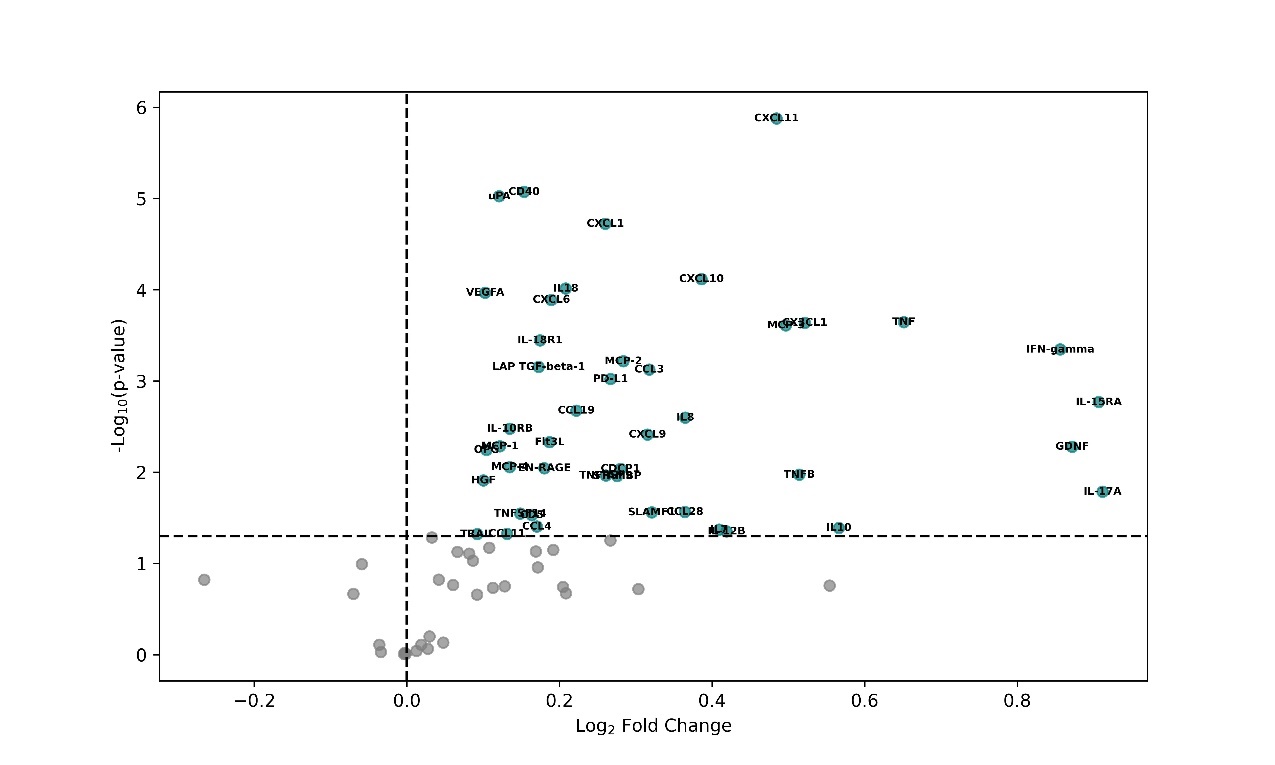


**Supplementary Fig. 1.** Comparison of inflammation-related proteins in candidemia subgroups. Volcano plot displays the log_2_ fold change against –log_10_ statistical p-value for proteins detected by the OLINK Inflammatory Panel in each candidemia subgroup compared to ICU-control subjects. (**A**) Panel, with n = 12 (C, isolated candidemia) over n = 20 (CON, control group), (**B**) Panel, with n = 12 (BC, candidemia with bacterial co-infection) over n=20 (CON, control group). The up- and down-regulated differentially expressed proteins (DEPs) are highlighted in green (adjusted p-value <0.05). Analysis based on NPX (Normalized Protein eXpression).
